# Artificial Intelligence for Molecular Biomarker Identification in Gastrointestinal and Hepatobiliary Cancers

**DOI:** 10.3390/ijms27156969

**Published:** 2026-08-03

**Authors:** Hyun-Jong Jang, Kwangil Yim, Sung Hak Lee

**Affiliations:** 1 Department of Physiology, College of Medicine, The Catholic University of Korea, Seoul 06591, Republic of Korea; hjjang@catholic.ac.kr; 2 Department of Hospital Pathology, Uijeongbu St. Mary’s Hospital, College of Medicine, The Catholic University of Korea, Uijeongbu 11765, Republic of Korea; kangse_manse@catholic.ac.kr; 3 Department of Hospital Pathology, Seoul St. Mary’s Hospital, College of Medicine, The Catholic University of Korea, Seoul 06591, Republic of Korea

**Keywords:** artificial intelligence, deep learning, digital pathology, gastrointestinal cancer, hepatobiliary cancer, molecular biomarker, precision oncology, whole-slide image

## Abstract

Artificial intelligence (AI) has emerged as a promising tool for inferring molecular biomarkers directly from digitized histopathologic slides. However, the current evidence in gastrointestinal and hepatobiliary cancers remains fragmented across tumor types and biomarker categories. Relevant studies were systematically identified and screened according to the PRISMA 2020 statement. PubMed, Embase, Web of Science Core Collection, Scopus, and IEEE Xplore were searched from 1 January 2015 to the date of search. Eligible studies involved gastrointestinal or hepatobiliary malignancies, used histopathology images, applied AI-based methods, and reported molecular biomarker prediction or inference. A total of 110 studies were included. Most studies focused on colorectal, gastric, liver, and pancreatic cancers, with microsatellite instability, mutation status, molecular subtypes, and tumor mutational burden being the most commonly investigated targets. Model architectures evolved from conventional convolutional neural networks to multiple-instance learning and transformer-based methods. While many studies reported promising predictive performance, direct comparison across studies remained challenging because of substantial heterogeneity in datasets, model architectures, and validation strategies. AI-based molecular biomarker identification from pathologic slides shows substantial promise in gastrointestinal and hepatobiliary cancers, but current evidence is constrained by heterogeneity and limited validation. Standardized, multicenter studies are needed before routine clinical implementation.

## 1. Introduction

Gastrointestinal and hepatobiliary cancers, including colorectal, gastric, hepatocellular, pancreatic, and biliary tract malignancies, represent a major global health burden and are among the leading causes of cancer-related morbidity and mortality worldwide [1]. These tumors exhibit substantial biological heterogeneity, and molecular characterization has become increasingly essential for diagnosis, prognostication, and therapeutic decision-making [2,3]. The identification of molecular biomarkers—such as gene mutations, microsatellite instability (MSI), gene expression signatures, and molecular subtypes—plays a central role in precision oncology, guiding targeted therapies and immunotherapy strategies [4]. Consequently, accurate and timely molecular characterization has become an integral component of contemporary cancer management.

Despite their clinical importance, conventional approaches for molecular biomarker assessment rely on techniques such as next-generation sequencing (NGS), polymerase chain reaction (PCR), and immunohistochemistry (IHC), which can be resource-intensive, time-consuming, and not universally accessible. Moreover, these assays often require additional tissue processing and can be limited by tissue availability, particularly in small biopsy specimens. As a result, there is growing interest in developing alternative or complementary approaches that can infer molecular information more efficiently from routinely available clinical data.

Histopathologic examination of hematoxylin and eosin (H&E)-stained slides remains the cornerstone of cancer diagnosis and is performed universally in clinical practice. With the widespread adoption of digital pathology and whole-slide images (WSIs), large-scale computational analysis of histologic images has become feasible. Emerging evidence suggests that subtle morphologic patterns present in histopathologic slides may reflect underlying molecular alterations, providing an opportunity to infer molecular phenotypes directly from image data [5].

In this context, artificial intelligence (AI), particularly deep learning (DL), has demonstrated remarkable performance in analyzing complex image data and has been increasingly applied to digital pathology [6,7]. Early studies primarily employed convolutional neural networks (CNNs) for patch-level classification tasks, whereas more recent approaches have leveraged multiple-instance learning (MIL), attention-based aggregation, and transformer-based architectures to capture slide-level representations under weak supervision. Furthermore, the emergence of foundation models trained on large-scale histopathology datasets has enabled the extraction of generalizable feature embeddings, which can be adapted for a wide range of downstream molecular prediction tasks [8].

A growing number of studies have explored the use of AI to predict molecular biomarkers from histopathologic images across various gastrointestinal and hepatobiliary cancers. These include prediction of MSI status in colorectal and gastric cancers, mutation status of key oncogenes and tumor suppressor genes (e.g., *KRAS*, *TP53*, *BRAF*), gene expression signatures, transcriptomic or molecular subtypes, and status of tumor mutational burden (TMB). However, the existing literature remains fragmented, with studies differing substantially in cancer types, biomarker targets, model architectures, datasets, validation strategies, and reported performance metrics. For example, although the area under the receiver operating characteristic curve (AUC) was the most commonly reported performance metric, some studies primarily reported accuracy, sensitivity, specificity, or other evaluation measures, limiting direct comparison across studies. Therefore, a comprehensive synthesis of the available evidence across biomarker categories is needed to better evaluate the maturity of the field, identify areas of robust evidence, and highlight gaps requiring further investigation.

Accordingly, in this review, we aimed to comprehensively evaluate studies applying AI to identify molecular biomarkers from histopathologic images in gastrointestinal and hepatobiliary cancers. We specifically organized the analysis according to biomarker categories, including mutation status, MSI, gene expression or transcriptomic signatures, molecular subtypes, and other categories of biomarkers. By synthesizing evidence across these categories and cancer types, we sought to provide a detailed overview of current methodological approaches, summarize model performance and validation strategies, and appraise the translational potential of this rapidly evolving field. These approaches raise the possibility that routine histopathology could serve not only as a diagnostic tool but also as a scalable surrogate for molecular profiling.

## 2. Methods

A systematic literature search was performed in PubMed, Embase, Web of Science Core Collection, Scopus, and IEEE Xplore. Search strategies were customized for each database using controlled vocabulary where available (e.g., MeSH in PubMed and Emtree in Embase) with free-text terms related to gastrointestinal and hepatobiliary cancers, histopathology or whole-slide imaging, artificial intelligence, and molecular biomarkers. The search period was restricted to studies published from 1 January 2015 through 31 March 2026, and only English-language original articles were considered. Search terms for each database are presented in the Supplementary File S1.

**Figure 1:**
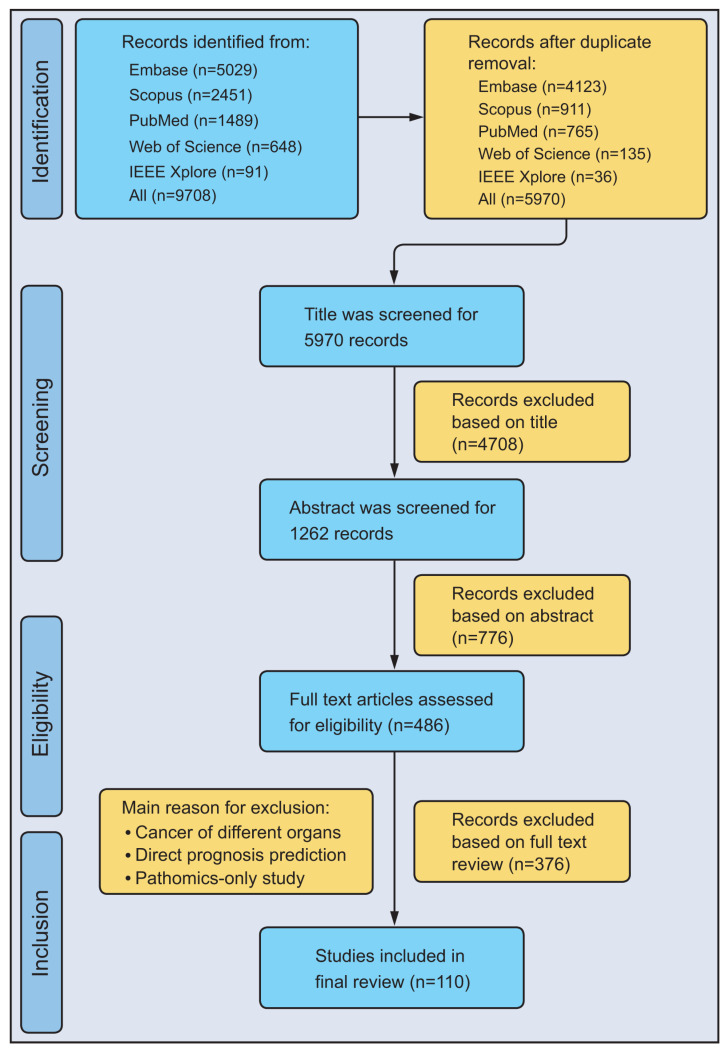
PRISMA 2020 flow diagram of the study selection process.

The study selection process was conducted in accordance with PRISMA 2020 guidelines (Figure 1). After removal of duplicate records, two reviewers independently screened titles and abstracts for eligibility. Full texts of potentially relevant studies were then assessed independently based on predefined inclusion and exclusion criteria (Table 1). Disagreements were resolved through discussion and consensus, with involvement of a third reviewer when necessary. Although a systematic search strategy and predefined eligibility criteria were applied, this study was designed as a narrative review with qualitative evidence synthesis and did not involve a formal meta-analysis. Therefore, prospective registration in PROSPERO was not undertaken.

The outcome of interest included model performance metrics and validation strategies. Data were extracted independently by two reviewers using a predefined standardized form to assess the outcome of the studies. Extracted variables included publication year, cancer type, cohort characteristics, sample size, molecular target, pathology image type, model architecture, validation strategy, performance metrics, and major findings.

The methodological quality and reporting transparency of the included studies were independently evaluated using the PROBAST and the TRIPOD+AI reporting guideline. PROBAST was used to assess the risk of bias across four domains: participants, predictors, outcomes, and analysis. Each domain was assessed as having low, high, or unclear risk of bias based on the adequacy of cohort selection, predictor acquisition, outcome definition, statistical analysis, validation strategy, and potential overfitting or generalizability concerns. An overall PROBAST judgment was subsequently assigned for each study according to the cumulative domain-level concerns.

Reporting quality was assessed using the TRIPOD+AI guideline, an extension of TRIPOD-2015 developed for AI-based prediction models. Reporting completeness and transparency were evaluated across key domains including study design, data acquisition and preprocessing, model development, validation, performance evaluation, reproducibility, and clinical applicability. Based on the overall percentage of adequately reported TRIPOD+AI items, studies were qualitatively categorized into low-to-moderate, moderate, moderate-to-high, or high adherence groups for comparative purposes. Because most included studies were retrospective DL studies using histopathological WSIs, several AI-specific methodological considerations were additionally examined during assessment, including patient-level data splitting, external validation using independent cohorts, robustness across institutions or specimen types, explainability analysis (e.g., attention heatmaps or Grad-CAM), and availability of source code or pretrained models. The distribution of studies according to risk of bias and reporting quality are shown in Supplementary Figure S1.

Given the substantial heterogeneity in cancer types, molecular targets, datasets, model architectures, and outcome measures, a quantitative meta-analysis was not considered appropriate. Therefore, findings were synthesized narratively and organized according to biomarker category, including mutation status, MSI, molecular subtypes, gene expression-related biomarkers, and other molecular features.

## 3. Results

A total of 110 studies met the predefined inclusion and exclusion criteria and were included in the final review (Figure 1). Studies were first categorized according to the molecular biomarker targeted by the AI model. The numbers of studies in each category were as follows: MSI prediction (n = 42), mutation prediction (n = 26), molecular subtype prediction (n = 19), treatment response or prognostic biomarker prediction (n = 17), TMB prediction (n = 8), IHC-based biomarker detection (n = 8), gene expression or transcriptomic signature prediction (n = 4), and other molecular biomarkers (n = 2). Because several studies evaluated more than one biomarker, the total number across categories exceeded 110. The distribution of studies according to cancer type was as follows: colorectal cancer (CRC, n = 60), gastric cancer (GC, n = 26), hepatocellular carcinoma (HCC, n = 9), pancreatic cancer (n = 7), gastrointestinal stromal tumor (n = 4), esophageal cancer (n = 3), biliary tract cancer (n = 1), intrahepatic cholangiocarcinoma (n = 1), and pan-cancer studies (n = 3).

The following sections highlight the key methodological contributions and unique findings of the included studies according to biomarker category. Corresponding tables provide detailed information on cancer type, target biomarker, training and validation cohorts, model architecture, and reported performance metrics. Within each biomarker category, studies are presented in chronological order according to their year of publication. If a study investigated multiple biomarkers, it was summarized only once under the biomarker category in which it was first discussed, whereas the corresponding table entry was included under each relevant biomarker category.

### 3.1. Microsatellite Instability

MSI is a molecular phenotype caused by defects in the DNA mismatch repair (MMR) system, leading to the accumulation of insertion and deletion mutations within repetitive DNA sequences known as microsatellites [9]. Based on the extent of microsatellite alterations, tumors are generally classified as microsatellite-stable (MSS) when no significant instability is detected, or as MSI-high (MSI-H) when instability is observed at multiple microsatellite loci. MSI is recognized as a key biomarker in gastrointestinal cancers, particularly CRC and GC, where it is associated with distinct clinicopathological characteristics and tumor biology. Furthermore, MSI has emerged as a predictive biomarker for response to immune checkpoint inhibitors (ICIs), making its accurate identification essential for precision oncology [10]. Consequently, the growing clinical importance of MSI has stimulated the development of AI-based approaches that aim to infer MSI status directly from routine histopathological images, potentially enabling rapid and cost-effective biomarker assessment. Included studies are briefly summarized by highlighting their distinctive methodological features and key findings, while detailed performance metrics are provided in Table 2.

Kather et al. (2019) developed a DL framework to predict MSI directly from H&E histology images across gastrointestinal cancers [11]. Using a ResNet18-based model, tumor regions were classified as MSS or MSI-H. The study demonstrated that the framework was generalizable across multiple cohorts and cancer types, suggesting its potential as a pan-cancer histomorphologic biomarker. This work laid the foundation for subsequent AI-based MSI prediction studies.

Echle et al. (2020) developed a clinical-grade DL model for detecting MSI and MMR deficiency (dMMR) in CRC directly from H&E WSIs [12]. Using a large international dataset of 8836 patients, the study demonstrated that large, heterogeneous training datasets are critical for developing generalizable models applicable across diverse clinicopathologic subgroups.

Krause et al. (2021) proposed a novel approach using conditional generative adversarial networks (CGANs) to generate synthetic histology images encoding MSI status in CRC [13]. The study showed that synthetic histology images preserve biologically meaningful information and can be effectively used for model training. This proof-of-concept highlights the potential of synthetic data to address data scarcity and privacy challenges in computational pathology.

Yamashita et al. (2021) developed MSINet, a DL model to predict MSI in CRC from H&E WSIs [14]. The study demonstrated that DL can support MSI screening and may reduce unnecessary molecular testing in clinical practice. In addition, the reader study suggested that AI-assisted prediction has the potential to complement conventional pathological assessment.

Bilal et al. (2021) developed a weakly supervised DL framework to predict molecular pathways (MSI, CIN, CIMP, hypermutation) and key mutations (*BRAF*, *TP53*, *KRAS*) directly from H&E WSIs in CRC [15]. The model employs an iterative draw-and-rank sampling strategy to identify highly informative tumor regions, enabling simultaneous prediction of multiple molecular alterations.

Muti et al. (2021) developed a DL framework to predict MSI and Epstein–Barr virus (EBV) status directly from H&E WSIs in GC using a large multicentre dataset [16]. The study showed that pooling data from multiple cohorts improved model generalizability across institutions. These findings emphasize the importance of large, heterogeneous training datasets for robust molecular prediction and support the potential use of DL as a pre-screening tool for immunotherapy-related biomarkers in GC.

Lee et al. (2021) proposed a fully automated DL pipeline for predicting MSI directly from H&E WSIs of CRC [17]. The framework consists of a sequential multi-stage pipeline, including (1) tissue vs. non-tissue filtering, (2) normal vs. tumor classification, and (3) MSI vs. MSS prediction using Inception-v3. The study demonstrated that expanding training data across institutions improves model generalizability, highlighting the importance of dataset diversity. The model showed potential as a screening tool, with the ability to pre-classify a substantial proportion (~40%) of cases without additional molecular testing.

Bustos et al. (2021) proposed xDEEP-MSI, a DL framework for predicting MSI in CRC from H&E images, with a particular focus on eliminating batch effects and dataset biases [18]. The model integrates tissue classification, multi-scale tiling, and an adversarial bias-rejecting architecture to minimize the influence of confounding factors, including project origin, patient-specific features, and TMA glass effects. This approach improved model generalizability by reducing dependence on dataset-specific biases.

Echle et al. (2022) developed and validated a DL-based system for detecting MSI/dMMR status directly from H&E CRC slides using a large multicentric dataset [19]. The model demonstrated clinical utility as a pre-screening tool, enabling up to ~52% of patients to be safely ruled out from further molecular testing at a fixed high sensitivity (95%).

Leiby et al. (2022) proposed an attention-based multiple instance learning (MIL) framework combined with self-supervised contrastive learning to predict MSI directly from H&E CRC WSIs [20]. The model leverages weak supervision to address label noise at the tile level and improves feature representation through contrastive learning.

Jiang et al. (2022) developed a DL model using MIL to predict MMR status in CRC directly from H&E WSIs [21]. The study proposed a dual-threshold triage strategy for rule-in/rule-out classification, enabling more efficient patient stratification while reducing unnecessary IHC testing.

Zhu et al. (2022) proposed an interpretable machine learning framework for predicting MSI from H&E WSIs in CRC and GC [22]. The study combines DL with a feature-based random forest model to improve model interpretability. Grad-CAM analysis highlighted diagnostically relevant regions, particularly immune cell-rich areas, providing insight into the histopathological features associated with MSI.

Schirris et al. (2022) proposed DeepSMILE, a weakly supervised DL framework that combines contrastive self-supervised learning with heterogeneity-aware MIL to predict MSI and homologous recombination deficiency (HRD) directly from H&E WSIs [23]. The method leverages self-supervised pretraining to learn domain-specific histopathology features without annotations and incorporates feature variability modeling to capture tumor heterogeneity.

Qiu et al. (2022) proposed a multimodal DL framework to predict MSI in CRC by integrating histopathological H&E images and multi-omics data [24]. The study analyzed molecular differences between MSI-H and MSS/MSI-L tumors and integrated histopathological image features with multi-omics data using multimodal compact bilinear pooling. The findings demonstrate the potential of multimodal learning to improve MSI prediction by combining complementary pathological and molecular information.

Su et al. (2022) developed an interpretable DL system to simultaneously predict tumor differentiation grade and MSI in GC directly from H&E WSIs [25]. The framework employs a two-stage pipeline consisting of tumor detection/differentiation followed by MSI prediction using tumor tiles. This study demonstrates the feasibility of simultaneously assessing histologic differentiation and molecular status within a single framework, supporting more integrated pathological evaluation.

Lou et al. (2022) proposed PPsNet, an improved DL framework for predicting MSI-H status in CRC directly from H&E WSIs [26]. The pipeline consists of two stages: tumor detection and MSI classification. For MSI prediction, the model incorporates pair-wise learning and synergic DL to reduce model complexity while addressing intra-class variability and inter-class similarity. The study demonstrates the potential of this strategy to improve the robustness and generalizability of MSI prediction using multicenter data.

Ding et al. (2022) proposed a spatially aware graph neural network framework to predict cross-level molecular profiles—including gene mutations, copy number alterations (CNA), MSI, and protein expression—directly from H&E WSIs in colon cancer [27]. The method transforms WSIs into graph-structured data by connecting image tiles based on their spatial relationships, enabling the modeling of tumor spatial heterogeneity. This graph-based approach demonstrates the feasibility of simultaneously predicting multiple molecular features while incorporating tissue spatial organization.

Schrammen et al. (2022) proposed SLAM (Slide-Level Assessment Model), a weakly supervised, annotation-free DL framework that simultaneously performs tumor detection and genotype prediction from CRC H&E WSIs [28]. Unlike prior pipelines that separate tumor localization and molecular prediction, SLAM integrates both tasks into a single end-to-end model using only slide-level labels. A key innovation is the introduction of a third “non-informative (normal tissue)” class, allowing the model to automatically exclude irrelevant regions and reduce noise from non-tumor tissue.

Lee et al. (2023) developed a fully automated DL pipeline to predict MSI from H&E WSIs in GC [29]. The model uses a multi-stage pipeline consisting of tissue detection, tumor classification, and MSI prediction based on Inception-v3. The study demonstrated that incorporating multicenter datasets improves model generalizability while highlighting the organ-specific nature of MSI-associated histomorphological features.

Rubinstein et al. (2023) developed a CNN-based framework to quantify tumor heterogeneity from H&E WSIs in colon cancer and investigated its association with MSI [30]. The model extracts deep imaging features and introduces a novel tumor heterogeneity score (THS) based on tile-level feature variability. The findings suggest that tumor heterogeneity derived from histopathology is associated with MSI and may provide complementary information for molecular characterization.

Gerwert et al. (2023) developed a label-free AI-based framework using infrared (IR) imaging of colon cancer tissues to predict MSI in without conventional staining [31]. The approach utilizes quantum cascade laser-based IR microscopy to capture molecular spectral information from unstained tissue sections, followed by a two-step DL pipeline for cancer detection and MSI classification. This study demonstrates the feasibility of stain-free molecular prediction, providing a potential alternative to conventional histopathological workflows.

Niehues et al. (2023) utilized DL for multiple biomarker prediction from H&E CRC histopathology slides [32]. The study demonstrated that self-supervised learning combined with attention-based MIL improves model generalizability across external cohorts. The framework showed promise for predicting MSI while illustrating the varying predictability of different molecular biomarkers.

Guo et al. (2023) developed a Swin Transformer-based DL framework to predict MSI and multiple molecular biomarkers (including hypermutation, CpG island methylator phenotype, chromosomal instability, and *BRAF*/*TP53* mutations) directly from H&E WSIs in CRC [33]. The study demonstrates that transformer-based architectures can outperform CNN-based models.

Yu et al. (2023) proposed a two-stage DL framework for predicting MSI in GC directly from H&E WSIs [34]. The pipeline first identifies tumor regions and subsequently classifies tumor patches into MSI vs. MSS. The model incorporates a residual attention network with non-local modules to capture both fine-grained local features and long-range spatial dependencies.

Wagner et al. (2023) developed a fully transformer-based DL pipeline for predicting key molecular biomarkers (MSI, *BRAF*, *KRAS*) directly from H&E WSIs in CRC [35]. Unlike prior CNN-based approaches, the model combines a pretrained transformer feature extractor with a transformer-based aggregation module to effectively capture global contextual relationships between image patches.

Zheng et al. (2024) developed an interpretable DL framework to predict dMMR/MSI-H status in GC from H&E WSIs [36]. The study employed StyleGAN-based latent space manipulation and CAM-guided blending to identify histopathological features associated with dMMR/MSI-H tumors. These explainable features also improved the diagnostic interpretation of junior pathologists, highlighting the potential of AI-assisted pathology.

Gustav et al. (2024) developed a transformer-based DL model to predict MSI directly from CRC histopathology and investigated whether the same model can also detect POLE mutations, a rare but clinically important hypermutated subtype [37]. The study demonstrated that the model could identify POLE-mutant tumors despite not being explicitly trained on POLE labels, suggesting shared histomorphological characteristics between MSI and POLE-mutant tumors.

Ciardiello et al. (2025) proposed an explainable DL framework for MSI detection in gastrointestinal cancers using H&E histopathology images [38]. The study compared multiple CNN architectures and a Vision Transformer (ViT) and incorporated CAM-based visualization together with robustness metrics to enhance model interpretability and reliability.

Gustav et al. (2025) developed a multi-target transformer-based DL model to simultaneously predict multiple biomarkers directly from H&E WSIs in CRC [39]. Using a large multicentre dataset, the study demonstrated the feasibility of simultaneous biomarker prediction within a single framework. This study highlights that genotype prediction from histology is heavily influenced by overlapping morphologic phenotypes.

Park et al. (2025) proposed MSI-SEER, a deep Gaussian process-based Bayesian model for predicting MSI directly from H&E WSIs in CRC and GC [40]. The model incorporates uncertainty estimation within a weakly supervised learning framework to improve prediction reliability. Furthermore, by integrating predicted MSI regions with tumor microenvironment (TME) features, the study demonstrated the potential to predict immunotherapy response in GC, extending its application beyond MSI detection.

Aldera et al. (2025) evaluated a transformer-based DL model for predicting MSI in CRC using H&E WSIs in a South African, ethnically heterogeneous cohort [41]. The study confirmed the generalizability of DL-based MSI prediction across diverse populations while demonstrating that model performance is influenced by population-specific tumor characteristics. These findings highlight the importance of regional calibration for clinical implementation.

Zhang et al. (2025) proposed a DL framework integrating histopathological image features and nuclear segmentation features to predict MSI and TMB from H&E WSIs in GC and CRC [42]. By combining complementary image and nuclear features, the study demonstrated the feasibility of simultaneously predicting multiple molecular biomarkers within a unified framework.

Feng et al. (2025) developed Deepath-MSI, a clinic-ready DL model for predicting MSI directly from H&E WSIs in CRC [43]. The model is based on a self-supervised pretrained feature extractor (DINOv2) and attention-based aggregation. Using a large multicenter dataset, the study demonstrated the clinical applicability of this framework while addressing limitations of earlier models in real-world settings.

El Nahhas et al. (2025) present STAMP (Solid Tumor Associative Modeling in Pathology), which provides a standardized, modular pipeline covering the entire process from problem definition to clinical translation [44]. The workflow consists of five stages: problem definition, data preprocessing, modeling, evaluation, and clinical interpretation, facilitating collaboration between clinicians and engineers. Designed as a biomarker-agnostic framework with support for multimodal inputs, STAMP provides a standardized foundation for the development and clinical translation of AI-based biomarker prediction models.

Bass et al. (2025) conducted a multi-centred blinded validation study of an AI-based biomarker test for predicting MSI/MMR status directly from H&E WSIs in CRC [45]. Using a large real-world multicenter dataset, the study demonstrated high concordance between the AI-based biomarker test and standard molecular testing.

Tang et al. (2025) developed a multimodal DL framework that integrates contrast-enhanced CT and WSIs to predict MSI in CRC [46]. The study developed pathology-based, radiology-based, and multimodal models using an adaptive residual learning strategy. The multimodal framework demonstrated the value of integrating complementary imaging modalities to improve the robustness and generalizability of MSI prediction.

Lee et al. (2025) proposed a multi-cancer DL framework for MSI prediction using H&E WSIs across colorectal (COAD/READ), gastric (STAD), and endometrial cancers (UCEC) from the TCGA dataset [47]. The study systematically compared tissue-specific, cross-tissue, and multi-tissue training strategies. The findings indicate that MSI-associated morphological features are partly tissue-specific while also supporting the existence of shared features that can enhance model generalizability across cancer types.

Yao et al. (2025) developed a multimodal DL framework integrating clinical variables, multiparametric MRI), and histopathology (H&E WSIs) to predict MSI in rectal cancer [48]. The authors developed clinical, radiomics, and pathomics models, followed by an integrated multimodal nomogram. This study demonstrates the potential of combining complementary clinical and imaging information to improve MSI prediction.

Salzano et al. (2026) evaluated the feasibility of general-purpose vision-language models (ChatGPT-4.0 and Gemini 2.5 Pro) for automated assessment of MMR status in CRC using IHC images for MMR proteins [49]. The study highlighted the potential of vision-language models for AI-assisted interpretation of MMR IHC while identifying the importance of internal positive controls for reliable assessment.

Petäinen et al. (2026) developed a DL framework to predict dMMR directly from CRC H&E WSIs by systematically evaluating tumor vs. non-tumor regions and multi-scale representations [50]. The study compared multiple modeling strategies using different magnifications, tissue regions, and foundation model-based embeddings combined with MIL architectures. The findings highlight the importance of multi-scale representations and demonstrate that both tumor and non-tumor regions, including the tumor microenvironment, contribute to dMMR prediction.

Tien et al. (2026) proposed Ensemble-MIL, a DL framework that integrates multiple MIL architectures to predict key CRC biomarkers (MSI-H, *BRAF*, *KRAS*) directly from H&E WSIs [51]. The pipeline consists of (1) tumor region detection, (2) self-supervised feature extraction, and (3) ensemble learning that combines attention-based, transformer-based, and graph-based MIL models. This ensemble strategy demonstrates the potential of integrating complementary MIL architectures for simultaneous biomarker prediction.

Wang et al. (2026) proposed ProMMF_Kron, a multimodal DL framework for predicting MSI from H&E WSIs in GC [52]. The model integrates histopathological images and gene expression data using a progressive multimodal fusion strategy, combining attention-based MIL image features with molecular features derived from differential gene expression analysis.

### 3.2. Mutational Status

The identification of somatic mutations in key oncogenes and tumor suppressor genes has become a cornerstone of precision oncology, as these genetic alterations drive tumor initiation, progression, and therapeutic response [53]. Therefore, the growing clinical demand for mutation profiling has stimulated the development of AI-based approaches capable of predicting genomic alterations directly from routine histopathological images, potentially reducing the need for additional molecular testing while expanding access to precision oncology (Table 3).

Chen et al. (2020) developed a DL model based on Inception-v3 to classify HCC histopathology and predict gene mutations directly from H&E WSIs [54]. The study demonstrated that a single framework could perform tumor classification, histologic grading, and mutation prediction, supporting the feasibility of inferring genomic alterations directly from routine histopathology.

Liao et al. (2020) developed a DL framework using H&E WSIs to perform both tumor classification and mutation prediction in HCC [55]. The CNN-based model demonstrated the feasibility of predicting multiple gene mutations from histopathology while showing partial generalizability in external validation.

Jang et al. (2020) developed a DL-based pipeline to predict clinically actionable gene mutations (*APC*, *KRAS*, *PIK3CA*, *SMAD4*, *TP53*) directly from H&E WSIs in CRC [56]. The framework employs a three-step patch-based pipeline, including tissue filtering, tumor region detection, and mutation classification using CNN architectures. The study demonstrated that incorporating multi-institutional datasets improves model generalizability, emphasizing the importance of dataset diversity for mutation prediction.

Bian et al. (2021) proposed ImmunoAIzer, a DL-based framework that simultaneously characterizes the TME and predicts gene mutations directly from H&E histopathology images of colon cancer [57]. The framework combines semi-supervised prediction of immune and tumor cell spatial distributions with mutation prediction from tumor regions.

Jang et al. (2021) proposed a fully automated DL pipeline to predict genetic mutations in GC directly from H&E WSIs [58]. The framework extracts tumor regions to classify mutation status for key genes (*CDH1*, *ERBB2*, *KRAS*, *PIK3CA*, *TP53*). The study demonstrated that histologic morphology contains predictive signals of genomic alterations while highlighting the importance of multi-institutional training for improving model generalizability. It also showed that mutation prediction is largely cancer type-specific, limiting cross-cancer model transferability.

Morel et al. (2022) developed a DL pipeline to predict mutational status directly from histopathology images across multiple cancers (breast, colorectal, lung, and ovarian) using TCGA data [59]. The study identified multiple clinically relevant mutations that could be inferred from histopathology and proposed three screening strategies (save-all, fixed-capacity, and prioritize) to optimize diagnostic workflows.

Ghareeb et al. (2022) developed a DL model to predict *KRAS* mutation status in rectal cancer directly from H&E histopathology images using multiple CNN architectures (MobileNet, ShuffleNet, Inception) [60]. The study compared different CNN architectures and demonstrated the feasibility of predicting *KRAS* mutations directly from routine histopathology.

Jiang et al. (2022) proposed a unified DL framework to perform three tasks in CRC: (1) tumor vs. normal tissue classification, (2) prediction of gene mutations, and (3) classification of consensus molecular subtypes (CMSs) [61]. The study demonstrated the feasibility of integrating multiple diagnostic tasks within a single framework, supporting comprehensive molecular characterization from routine histopathology.

Fu et al. (2023) developed a DL model to predict both prognosis and mutation status directly from H&E WSIs in gastrointestinal stromal tumors [62]. Using a large multicenter cohort, the study demonstrated the potential of a framework for prognostic stratification and mutation prediction. The model also identified clinically relevant mutations associated with therapeutic response, supporting its potential application in precision oncology.

Wei et al. (2023) developed a DL-based framework to predict multiple immunotherapy-related molecular features from H&E WSIs in GC, including immunotherapy-sensitive subtypes (EBV, MSI, TMB, PD-L1), gene mutations (*KRAS*, *PIK3CA*, *TP53*, *MUC16*), and pathway activity [63]. The study demonstrated the feasibility of predicting diverse immunotherapy-related biomarkers within a unified framework. Additional segmentation analysis further revealed associations between immunotherapy-sensitive subtypes and inflammatory as well as stromal cell components.

Chen et al. (2023) proposed an unsupervised clustering-optimized MIL framework for predicting gene mutations directly from H&E WSIs [64]. Instead of using all image patches or restricting analysis to tumor regions, the model applies K-means clustering to group patches into distinct tissue phenotypes and selects the most informative clusters for mutation prediction.

Saldanha et al. (2023) conducted a large-scale study to evaluate pan-cancer mutation prediction from histopathology using a DL pipeline that integrates self-supervised feature extraction and attention-based MIL [65]. The authors systematically assessed the predictability of clinically relevant gene mutations across multiple tumor types. The study demonstrated that the feasibility of mutation prediction varies across cancer types, reflecting differences in the histomorphological manifestations of genomic alterations.

Li et al. (2024) developed a DL framework to predict *KRAS*, *NRAS*, and *BRAF*V600E mutation status simultaneously from H&E WSIs in left-sided CRC [66]. The model combines CNN-based patch-level prediction with MIL-based slide-level aggregation and further integrates clinicopathological information. This multimodal strategy highlights the potential of combining histopathological and clinical features for mutation prediction.

Singh et al. (2024) proposed KRASFormer, a fully ViT-based framework for predicting *KRAS* gene mutations directly from H&E WSIs in CRC [67]. The study demonstrates the feasibility of applying transformer-based architectures to mutation prediction directly from routine histopathology.

Xiao et al. (2025) developed a histopathology-based AI system to predict actionable genetic alterations in intrahepatic cholangiocarcinoma directly from H&E WSIs [68]. Using a CNN-based MIL framework with self-supervised learning, the model predicted key genetic alterations such as *FGFR2* and *IDH* with moderate performance.

Zhou et al. (2025) developed a DL-assisted digital pathology framework to optimize *BRAF* V600E IHC interpretation criteria in CRC [69]. The model quantified IHC features and established a clinically relevant cutoff that improved concordance with genetic testing.

Bonetti et al. (2026) conducted a large-scale multicenter, multinational study to predict molecular mutations (*KIT*, *PDGFRA*), treatment sensitivity, and recurrence-free survival (RFS) in gastrointestinal stromal tumors directly from H&E WSIs using DL [70]. The authors utilized a foundation model-based pipeline for mutation, treatment response, and outcome prediction. The study demonstrates the potential of foundation model-based AI to support comprehensive molecular characterization and therapeutic decision-making from routine histopathology.

Zhang et al. (2026) tried to predict both clinically relevant gene mutations (*KRAS*, *NRAS*, *BRAF*) from H&E WSIs and HER2 expression from IHC WSIs in CRC [71]. They leveraged ViT-based feature extraction (UNI) combined with attention-based MIL (CLAM) to aggregate patch-level information into slide-level predictions.

Wang et al. (2026) proposed TEWS (Transformer-empowered weakly supervised model) for predicting immune scores and genetic mutations directly from WSIs in HCC [72]. The transformer-based architecture captures both local histologic features and global contextual information, addressing limitations of conventional CNN-based approaches. The study highlights the potential of transformer-based weakly supervised learning for integrated prediction of immune and molecular biomarkers from routine histopathology.

### 3.3. Molecular Subtype

Molecular subtypes provide a biologically meaningful framework for categorizing tumors beyond individual genetic alterations by integrating diverse molecular characteristics, including gene expression patterns, signaling pathways, and features of the TME. Accurate identification of molecular subtypes has become increasingly important for advancing precision oncology and guiding individualized patient management [73]. This section also covers other biologically and clinically relevant cancer subtypes, including immune subtypes. Furthermore, EBV-positive GC is recognized as a distinct molecular subtype with unique genomic, epigenetic, and immune features. Accordingly, studies aimed at predicting EBV status from histopathological images are also reviewed in this section (Table 4).

Liang et al. (2021) developed CNN-based models to predict drug-sensitive mutation subtype (*KIT* exon 11 mutation) and other types (*KIT* exon 9 mutations, *KIT* other mutations, *PDGFRA* mutations, and wild-type) in gastrointestinal stromal tumors directly from H&E WSIs [74]. Using a multicenter dataset, the study demonstrated the feasibility of predicting clinically relevant mutation subtypes directly from routine histopathology.

Hinata et al. (2021) developed a DL model to detect immunotherapy-sensitive GC subtypes (EBV and MSI/dMMR) directly from H&E WSIs [75]. The model was trained to classify EBV-positive and MSI/dMMR tumors jointly by leveraging their shared histologic characteristics. Interpretability analysis identified intraepithelial lymphocytosis as a key morphological feature, supporting the biological relevance of the model.

Sirinukunwattana et al. (2021) developed an image-based DL framework (imCMS) to predict CMS of CRC directly from H&E histopathology images [76]. Importantly, imCMS captured intratumoural heterogeneity at the tile level and successfully classified samples that were previously unclassifiable by RNA-based methods, demonstrating the complementary value of image-based molecular subtyping.

Chen et al. (2021) proposed a framework to define immune subtypes of GC based on tumor-infiltrating immune cells and to predict these subtypes directly from WSIs using DL [77]. Using immune cell profiles derived from transcriptomic data, the study identified reproducible immune subtypes and developed a CNN-based model for their prediction from histopathology.

Zheng et al. (2022) proposed EBVNet, a DL model to predict EBV status in GC from histopathology images [78]. Interpretability analyses showed that the model captured established histopathological features of EBV-associated GC, while a human–AI collaborative approach further enhanced diagnostic interpretation.

Jeong et al. (2022) developed another EBVNet, a DL framework to predict EBV status in GC from H&E WSIs [79]. The study demonstrated the feasibility of automated EBV prediction from routine histopathology and highlighted its potential to support pathologists in identifying EBV-associated gastric cancer.

Vuong et al. (2022) developed a DL-based classifier to predict EBV status in GC directly from H&E WSIs, with a particular focus on biopsy specimens [80]. The study developed an EfficientNet-based framework that enables EBV prediction from biopsy specimens while providing spatial visualization of EBV-associated regions.

Flinner et al. (2022) developed a DL-based model to predict TCGA molecular subtypes (EBV, MSI, GS, CIN) of GC directly from H&E WSIs [81]. The study demonstrated the feasibility of molecular subtyping from routine histopathology using an ensemble CNN framework and identified subtype-specific histopathological features. It also highlighted intratumoral heterogeneity, suggesting that multiple molecular subtypes may coexist within a single tumor.

Wang et al. (2022) proposed DEMoS, a DL-based ensemble framework to predict TCGA molecular subtypes of GC directly from H&E WSIs [82]. The model integrates multiple EfficientNet classifiers through an ensemble strategy to improve robustness and reduce overfitting.

Saillard et al. (2023) developed a multi-step DL framework to predict molecular subtypes of pancreatic ductal adenocarcinoma directly from H&E WSIs and to characterize intratumor heterogeneity at both slide and tile levels [83]. The pipeline sequentially performs tumor detection, tumor–stroma classification, and subtype prediction. The study demonstrated the clinical value of characterizing intratumoral heterogeneity and showed that aggressive basal-like components have important prognostic implications.

Ahmadvand et al. (2024) developed a DL model to classify molecular subtypes (classical vs. basal-like) of pancreatic ductal adenocarcinoma from H&E WSIs [84]. A two-stage pipeline was adopted, consisting of tumor region detection followed by subtype classification. Interpretability analysis identified gland formation as a key histopathological feature for subtype discrimination, supporting the biological relevance of the model.

Hyeon et al. (2024) developed a DL framework to predict chromosomal instability (CIN) in CRC directly from H&E WSIs [85]. The study combined tile-level CNN features with nucleus-level morphological features to improve CIN prediction. The findings further demonstrated that nuclear morphological variability is closely associated with CIN, supporting the use of quantitative morphologic features as surrogate biomarkers.

Lafarge et al. (2024) developed an image-based consensus molecular subtyping (imCMS) framework to classify rectal cancer into CMSs (CMS1–4) directly from H&E WSIs [86]. Using a multicentric dataset with paired transcriptomic data, the authors trained the model on both resection and biopsy specimens to improve generalizability across specimen types. The study demonstrated that imCMS is associated with treatment response, supporting its potential use for therapeutic stratification.

Byeon et al. (2025) developed a DL-based framework (EBV-TRACER) to predict EBV status directly from H&E GC slides [87]. The model integrates a two-stage stain normalization strategy with an EfficientNet-based classifier and patch-to-slide aggregation to improve robustness across biopsy and resection specimens.

Kaprio et al. (2025) developed an IHC-based classification system for CRC that approximates CMS using CNNs for automated staining interpretation [88]. By integrating conventional IHC markers with β-catenin and AI-assisted quantification, the study demonstrated the feasibility of image-based CMS classification and its potential for overall survival (OS) stratification.

Seraphin et al. (2025) developed a transformer-based DL model to predict outcome-related molecular subclasses (S1/S2 vs. S3) and microvascular invasion (mVI) directly from H&E WSIs in HCC [89]. The transformer-based framework predicted molecular subclasses and mVI while capturing biologically meaningful TME characteristics. Importantly, AI-predicted molecular subclasses and mVI status were significantly associated with OS.

Le Douget et al. (2025) developed a DL framework to predict CMS and intratumor heterogeneity in colon cancer using H&E WSIs [90]. Using large multicohort datasets, the study demonstrated that DL-based histology can approximate transcriptomic CMS classification while enabling spatial mapping of intratumoral heterogeneity. The findings further showed that CMS heterogeneity is associated with patient prognosis, highlighting the clinical value of image-based molecular subtyping.

Akbar et al. (2026) developed PanSubNet, a DL framework to predict molecular subtypes (basal-like vs. classical) of pancreatic ductal adenocarcinoma directly from H&E WSIs [91]. The model integrates multi-scale representations by combining cell-level and tissue-level features through an attention-based fusion mechanism. The study demonstrated robust generalizability across institutions and showed that histology-based molecular subtyping can provide prognostic information beyond transcriptomic classification.

### 3.4. Treatment Response or Prognostic Biomarker

While most studies have focused on predicting specific molecular biomarkers, increasing attention has been directed toward the identification of image-derived surrogate biomarkers associated with treatment response and clinical outcomes. These biomarkers are based on morphological features embedded within histopathological images that may reflect complex molecular, cellular, and microenvironmental processes underlying tumor behavior. DL enables the extraction of such latent information from WSIs and facilitates the identification of biomarkers that may inform therapeutic sensitivity and prognostic stratification without additional molecular testing. This section summarizes studies investigating clinically relevant surrogate biomarkers linked to treatment response and prognosis in gastrointestinal and hepatobiliary cancers (Table 5).

Zhao et al. (2020) developed a CNN-based model for fully automated quantification of tumor-stroma ratio (TSR) from H&E WSIs in CRC [92]. The model performs multi-class tissue segmentation and computes TSR at the whole-slide level. The study demonstrated that AI-derived TSR is an independent prognostic biomarker and that incorporating TSR into prognostic models improves risk stratification beyond conventional clinicopathological factors.

Patil et al. (2023) developed a DL model to quantify reticulin proportionate area (RPA) from reticulin-stained WSIs in HCC [93]. The study demonstrated that AI-derived RPA reflects aggressive tumor behavior and provides independent prognostic information beyond conventional clinicopathologic variables.

Makhlouf et al. (2023) proposed True-T, an AI-based pipeline for quantifying T-cell immune response from IHC images in CRC [94]. The framework consists of a three-stage pipeline that performs T-cell segmentation, cell density estimation, and survival prediction based on CD3, CD4, and CD8 densities. This approach demonstrates the potential of AI-assisted immune profiling for prognostic stratification and shows its applicability across multiple cancer types.

Zeng et al. (2023) developed a DL model (ABRS-P) to predict the atezolizumab–bevacizumab response gene signature (ABRS) directly from H&E WSIs in HCC [95]. Using a CLAM-based MIL framework, the model predicted a transcriptomics-derived immunotherapy response signature from routine histopathology. The study further demonstrated that AI-predicted response signatures correspond to immune-active tumor microenvironments, supporting their potential use for treatment selection.

Nimgaonkar et al. (2023) developed an AI-derived histologic signature to predict response to adjuvant gemcitabine in pancreatic ductal adenocarcinoma using H&E WSIs [96]. The model integrates nuclei segmentation with quantitative morphologic feature extraction to identify treatment-related histologic signatures. The study demonstrated the potential of AI-derived histologic biomarkers for predicting treatment response, providing complementary information beyond RNA-based molecular subtyping.

Horst et al. (2023) developed DL models to predict response to neoadjuvant chemotherapy in esophageal and gastroesophageal junction adenocarcinoma using H&E WSIs [97]. The study demonstrated that pretreatment histopathological features contain predictive information for chemotherapy response, supporting AI-assisted treatment stratification.

Tian et al. (2024) developed a weakly supervised DL framework integrating masked autoencoders with ResNet to predict M2 macrophage abundance directly from H&E WSIs in HCC [98]. The study demonstrated that AI-derived M2 macrophage levels are associated with patient prognosis and that integrating AI-derived features with clinical variables improves prognostic assessment.

Liu et al. (2024) developed ICIsNet, a DL-based digital pathology model to predict response to first-line PD-1 inhibitor + chemotherapy in advanced GC using pre-treatment H&E WSIs [99]. The model combines EfficientNet-B4, DenseNet121, and Swin Transformer V2 within an ensemble framework to generate a quantitative biomarker (Immune Checkpoint Inhibitor Response Score). This framework demonstrates the potential of AI-assisted pathology for identifying patients likely to benefit from immunotherapy.

Kim et al. (2025) investigated an AI-powered HER2 quantification approach using HER2 IHC WSIs to refine HER2 positivity criteria and predict treatment response in HER2-positive biliary tract cancer treated with trastuzumab plus folinic acid, fluorouracil, and oxaliplatin (FOLFOX) [100]. The study demonstrated that AI-derived HER2 quantification improves treatment stratification beyond conventional assessment and revealed distinct spatial immune phenotypes associated with HER2 expression.

Kong et al. (2025) developed a weakly supervised DL model to predict tyrosine kinase inhibitor (TKI) response directly from H&E histopathology in gastrointestinal stromal tumors [101]. The study demonstrated that histology-based AI can predict treatment sensitivity directly from routine pathology and may capture functional drug response beyond genomic alterations.

Wu et al. (2025) developed a DL framework to predict both HER2 status and trastuzumab treatment response directly from H&E WSIs in GC [102]. Using a CLAM-based MIL framework, the study demonstrated the feasibility of predicting HER2 status and treatment response from routine histopathology.

Zhu et al. (2026) developed a pathomics-based DL framework to predict response to neoadjuvant chemoimmunotherapy in esophageal squamous cell carcinoma [103]. Using H&E WSIs, the model segmented tissue into tumor, stroma, lymphocyte, and necrotic regions and extracted spatially weighted pathomic features to construct a pathomic signature. This signature was integrated with clinical variables to improve treatment response prediction, highlighting the complementary value of combining histopathological and clinical information.

Hendifar et al. (2026) developed a computational histology-based AI biomarker using the CHAI platform to predict optimal first-line chemotherapy in advanced pancreatic ductal adenocarcinoma from H&E WSIs [104]. The model extracts quantitative histomorphologic features to stratify patients based on their predicted benefit from fluoropyrimidine- or gemcitabine-based therapy. The study demonstrated that histology-based AI provides predictive information independent of RNA-based subtypes, supporting its potential for personalized treatment selection.

### 3.5. Tumor Mutational Burden

TMB is a quantitative measure of the number of somatic mutations present within a tumor genome and serves as an indicator of tumor immunogenicity. High TMB is associated with increased neoantigen generation and has emerged as a predictive biomarker for response to ICIs across multiple cancer types [105]. Consequently, accurate assessment of TMB has become increasingly important for patient stratification and treatment selection in precision oncology (Table 6).

Zhang et al. (2019) explored the feasibility of predicting TMB directly from HCC histopathological images using DL [106]. Because conventional CNN architectures suffered from substantial overfitting, the authors developed a customized CNN optimized for capturing fine-grained histologic patterns. The study highlights the importance of tailoring network architecture to the morphological characteristics of histopathology for TMB prediction.

Wang et al. (2020) proposed a DL framework to predict TMB directly from H&E WSIs in gastrointestinal cancers [107]. Using transfer learning with multiple CNN architectures and an MIL strategy, the model classified patients into high- and low-TMB groups. This study demonstrated the feasibility of predicting TMB directly from routine histopathology across multiple gastrointestinal cancer types.

Shimada et al. (2021) investigated whether TMB-high (TMB-H) CRC can be predicted directly from H&E histology using DL [108]. Using digital pathology datasets from Japanese CRC and TCGA cohorts, the authors developed a CNN-based pipeline with tile-level analysis for TMB prediction.

Huang et al. (2022) proposed a multi-modal DL framework to predict TMB status in CRC directly from H&E WSIs combined with clinical data [109]. The model integrates CNN-derived image features with clinical variables through multimodal feature fusion. The study demonstrated that combining histopathological and clinical information enhances TMB prediction compared with image-based analysis alone.

Liu et al. (2022) proposed a DL framework (DeepHE) to predict TMB directly from H&E WSIs in CRC [110]. The study demonstrated the feasibility of TMB prediction from routine histopathology while highlighting the need for external validation to establish model generalizability.

Li et al. (2024) proposed a multimodal DL framework to predict TMB in GC by integrating histopathological images, clinical variables, and multi-omics data [111]. The framework combines CNN-derived image features with selected clinical and molecular features through multimodal integration. The study demonstrated the complementary value of integrating histopathology with multi-omics data for TMB prediction.

Wang et al. (2025) proposed an ensemble transformer-based MIL framework (ETMIL-SSLViT) to predict both TMB and pathological subtypes directly from H&E WSIs in endometrial cancer (EC) and CRC [112]. The transformer-based framework enabled effective modeling of inter-patch relationships and global tissue context through an ensemble MIL strategy.

### 3.6. Immunohistochemistry-Based Biomarker

Many molecular biomarkers used in clinical practice are identified through immunohistochemical assessment. Recent studies have explored the application of DL for automated quantification of biomarker expression in IHC-stained slides as well as the prediction of IHC-derived biomarker status directly from H&E images. This section summarizes studies investigating AI-based approaches for IHC biomarker assessment in gastrointestinal and hepatobiliary cancers (Table 7).

Sarker et al. (2021) developed a U-Net-based workflow for automated detection and quantification of ICOS protein expression in CRC using IHC WSIs [113]. The study demonstrated that AI-derived ICOS quantification is associated with patient prognosis, highlighting the potential of automated biomarker assessment for prognostic stratification.

Han et al. (2022) proposed a DL-based automatic HER2 scoring framework for GC using HER2 IHC WSIs [114]. The model consists of a tile-level classifier and a WSI-level prediction network, enabling fully automated HER2 scoring without manual region annotation.

Wang et al. (2025) developed a DL-based AI-IHC pipeline to predict multiple clinically relevant biomarkers directly from H&E WSIs in gastrointestinal cancers [115]. Using an automated annotation pipeline and a semi-supervised learning framework, the authors developed virtual models for multiple IHC biomarkers (Pan-CK, P40, Desmin, P53, and Ki-67) from routine H&E slides. This study demonstrates the potential of virtual staining to complement or reduce the need for conventional IHC in biomarker assessment.

Liao et al. (2025) developed HER2Net, a DL framework to predict HER2 status in GC directly from H&E WSIs [116]. Unlike conventional binary classification approaches, the model quantitatively estimates the proportion of HER2 high-expression regions at the pixel level.

Fischer et al. (2026) proposed a contrastive virtual staining framework that generates synthetic IHC images (*KRT81*, *HNF1A*) from routine H&E slides for pancreatic ductal adenocarcinoma subtyping [117]. The framework introduces a semipaired dataset and a contrastive-inspired CycleGAN to improve virtual staining by incorporating anatomical correspondence between adjacent tissue sections.

### 3.7. Gene Expression or Transcriptomic Signature/Other Biomarkers

Gene expression profiles and transcriptomic signatures provide insights into the functional state of tumors by capturing the activity of biological pathways, cellular processes, and interactions within the TME [118]. These signatures have demonstrated clinical utility for molecular classification, prognostic stratification, and prediction of therapeutic response across multiple cancer types (Table 8).

Schmauch et al. (2020) proposed HE2RNA, a DL model to predict genome-wide RNA-Seq expression directly from H&E WSIs across 28 cancer types in TCGA [119]. The model predicted transcriptomic profiles and captured biologically relevant pathways, particularly immune-related signatures. The study further demonstrated that the learned transcriptomic representation can be transferred to downstream molecular prediction tasks, highlighting its potential as a generalizable molecular feature extractor.

Zeng et al. (2022) developed DL models to predict the activation of immune and inflammatory gene signatures directly from H&E WSIs in HCC [120]. Using TCGA as a discovery cohort and an external hospital cohort for validation, the study compared patch-based, MIL, and CLAM approaches. The findings demonstrated the generalizability of AI-based gene signature prediction across different staining protocols and transcriptomic platforms.

El Nahhas et al. (2024) proposed a regression-based DL framework (CAMIL regression) for predicting continuous molecular biomarkers directly from H&E WSIs across multiple cancer types [121]. The framework predicts HRD and multiple TME biomarkers using a regression-based approach. The study demonstrated the advantages of regression-based biomarker prediction for capturing molecular variation across diverse cancer types.

Nonchev et al. (2025) proposed DeepSpot, a DL framework that predicts spatial transcriptomics directly from H&E WSIs by leveraging pathology foundation models and multi-level spatial tissue context [122]. The model integrates fine-grained cellular features with tissue-level organization to infer spatial gene expression patterns. The study demonstrated the feasibility of predicting transcriptomic profiles directly from routine histopathology and highlighted their utility for downstream clinical applications.

Nonchev et al. (2025) proposed DeepSpot2Cell, a DL framework that predicts virtual single-cell spatial transcriptomics from H&E images using only spot-level supervision [123]. The model extends the DeepSpot architecture by integrating cell-level, spot-level, and neighboring spatial context to generate virtual single-cell expression profiles. The model enables accurate deconvolution of spot-level data into cell-level expression profiles, providing a scalable approach to generate virtual single-cell transcriptomics from routine histology images.

Aalam et al. (2025) proposed OncoMet, a DL-based framework for predicting metastatic risk and oncogenic signaling pathway activation directly from H&E WSIs in esophageal cancer [124]. The model combines deep feature extraction with slide-level prediction to infer metastatic potential and the activation of key oncogenic pathways. The study demonstrates the potential of AI-assisted pathology to predict tumor behavior and underlying molecular signaling from routine histopathology.

## 4. Discussion

This review summarizes the rapidly expanding body of literature investigating AI-based prediction of molecular biomarkers from histopathological images in gastrointestinal and hepatobiliary cancers. While previous reviews have discussed the broader application of AI in computational pathology and the emerging role of AI-derived molecular biomarkers [6,125,126], most were published before the recent expansion of transformer-based architectures, foundation models, multimodal approaches, and large-scale multicenter validation studies. By including studies published through March 2026, the present review provides an updated and comprehensive overview of AI-based molecular biomarker identification in gastrointestinal and hepatobiliary cancers. Furthermore, studies were systematically categorized according to biomarker type, and methodological quality and reporting transparency were evaluated using PROBAST and TRIPOD+AI. Across 110 included studies, MSI, mutation status, molecular subtypes, TMB, gene expression signatures, and other clinically relevant biomarkers were explored using increasingly sophisticated DL approaches. Collectively, these studies support the concept that routinely acquired histopathological images contain latent molecular information that can be extracted by AI models and potentially utilized for molecular characterization.

However, although overall trends in predictive performance can be identified across biomarker categories, systematic comparison of model performance across studies remains challenging. Considerable heterogeneity was observed in cohort composition, sample size, specimen type, scanner platforms, model architectures, validation strategies, and reported performance metrics. Furthermore, studies differed substantially in the use of internal validation, cross-validation, external validation, and multicenter testing. As a result, direct comparison of reported AUCs between studies should be interpreted cautiously, and apparent performance differences may not necessarily reflect superiority of a particular model architecture.

Another important consideration when interpreting model performance across studies is that the reported AUC values are influenced not only by differences in validation strategies but also by variations in case mix, disease prevalence, and cohort composition. Studies conducted in highly selected datasets may report higher discriminative performance than those evaluating more heterogeneous real-world populations. In addition, differences in specimen type, disease stage distribution, and institutional characteristics can affect model performance and limit direct comparison across studies. Furthermore, models with similar AUCs may differ substantially in their clinical usefulness depending on the thresholds selected for decision-making. Decision curve analysis can provide additional insight into the potential net clinical benefit of implementing AI-based biomarker prediction models in practice. However, only a limited number of studies included calibration assessments or decision-analytic evaluations. Future studies should therefore adopt more comprehensive reporting frameworks in addition to AUC. Such efforts would facilitate more meaningful comparisons across studies and better support the translation of AI models into clinical workflows.

One of the most notable findings of this review is the relative maturity of MSI prediction compared with other biomarker categories. MSI was the most extensively investigated biomarker and consistently demonstrated the highest predictive performance across multiple studies and independent cohorts. Several studies reported AUCs exceeding 0.90 and further proposed clinically applicable screening strategies capable of reducing unnecessary molecular testing while maintaining high sensitivity for MSI detection [14,17,19,21]. These findings suggest that MSI prediction is currently the closest AI-based molecular biomarker application to potential clinical implementation. In contrast, although promising results have been reported for other biomarkers, performance remains more variable and generally insufficient for routine clinical use as a stand-alone screening tool. These findings suggest that the predictability of molecular biomarkers from histology is highly dependent on the extent to which the underlying molecular event manifests as recognizable tissue-level phenotypes.

Importantly, biomarker prediction models that have not yet achieved sufficient performance for clinical screening purposes may still provide meaningful clinical value. Several studies demonstrated that AI-derived biomarker predictions were independently associated with clinical outcomes even after adjustment for conventional clinicopathological variables [90,91]. This suggests that AI models may capture latent biological information that is not fully represented by existing molecular assays or routine pathological assessment. Therefore, even when image-based biomarker prediction cannot replace molecular testing, it may still provide complementary prognostic information and contribute to risk stratification frameworks.

From a methodological perspective, a clear evolution was observed across the included studies. Early studies predominantly relied on CNNs, whereas more recent investigations increasingly adopted MIL, attention-based aggregation methods, transformer architectures, and pathology foundation models. These approaches are particularly attractive because they facilitate learning from weakly labeled WSIs while capturing long-range spatial relationships and complex tissue heterogeneity. Foundation models pretrained on large histopathology datasets may further improve generalizability and data efficiency, potentially accelerating future biomarker discovery efforts.

Multimodal approaches represent one of the most promising future directions for improving biomarker prediction performance. Studies integrating histopathological images with radiological imaging, genomic data, transcriptomic profiles, or clinical variables generally reported superior performance compared with image-only models. Because cancer biology is inherently multifactorial, it is unlikely that any single data modality can fully capture the complexity of tumor behavior. Future development of multimodal AI systems capable of integrating pathological, molecular, radiological, and clinical information may therefore offer the greatest opportunity for clinically meaningful precision oncology applications.

This review has several limitations. First, although a comprehensive literature search was performed, relevant studies may have been missed because only English-language full-text publications indexed in the selected databases were included. Second, the substantial heterogeneity in study design, patient cohorts, biomarker definitions, AI architectures, and validation strategies precluded direct quantitative comparisons or meta-analysis. Finally, because many included studies were retrospective and single-center studies, the reported performance may not fully reflect real-world clinical applicability. Prospective multicenter studies using standardized reporting and external validation will be essential to facilitate robust comparison and clinical translation.

Another potential limitation of the current evidence base is publication bias. Studies reporting strong predictive performance are generally more likely to be published than those with negative or inconclusive results. Consequently, the published literature may overestimate the true performance and generalizability of AI-based biomarker prediction models. This concern is particularly relevant in rapidly evolving fields such as AI, where novel and high-performing models tend to receive greater attention. Future efforts to promote transparent reporting and publication of negative findings will be important for obtaining a more balanced assessment of model performance.

## 5. Conclusions

In conclusion, AI-based molecular biomarker prediction from histopathological images has demonstrated substantial potential in gastrointestinal and hepatobiliary cancers. Among currently available applications, MSI prediction appears to be the most mature and clinically translatable, whereas other biomarker categories remain at earlier stages of development. Continued advances in model architecture, foundation models, multimodal learning, and large-scale external validation will be essential to achieve reliable clinical implementation and to fully realize the potential of computational pathology as a surrogate platform for molecular characterization.

## Figures and Tables

**Table 1:** Inclusion and exclusion criteria for study selection.

Category	Inclusion Criteria	Exclusion Criteria
**Population**	Patients with gastrointestinal or hepatobiliary cancers (e.g., colorectal, gastric, liver, pancreatic, biliary tract cancers)	Not gastrointestinal or hepatobiliary cancers (e.g., lung, breast, prostate, hematologic malignancies)
**Data Modality**	Studies using histopathology images, including whole-slide images, digital pathology, or tissue slide images	Studies using radiology only (CT, MRI, PET), endoscopy images without histology, omics-only studies without pathology images, pathomics-only studies without AI-based feature extraction
**Intervention Method**	Studies applying artificial intelligence methods, including deep learning, machine learning, CNNs, transformers, or foundation models	Studies without AI methods (e.g., conventional statistical analysis only), purely descriptive pathology studies
**Outcome**	Prediction or inference of molecular biomarkers, including mutation status, gene expression, molecular subtypes, MSI, or omics-related features, etc.	Studies focusing only on survival, prognosis, grading, or classification without molecular biomarker prediction
**Study Design**	Original research articles (retrospective or prospective studies)	Reviews, meta-analyses, editorials, letters, conference abstracts, case reports
**Language**	Published in English	Non-English publications
**Subjects**	Human studies	Animal studies, in vitro-only studies
**Availability**	Full text available	Abstract-only studies
**Publication Date**	Published from 1 January 2015 onward	Studies published before 2015

**Table 2:** Characteristics and performance of studies predicting microsatellite instability from histopathological images. Abbreviations are defined in the main text. Case numbers are reported as slide counts (n) or patient counts (N), as specified in each study.

Reference	Cancer Type	Target	Training/Validation Cohort	Base Model	Performance Measure
Kather et al. (2019) [11]	Colorectal, gastric, and endometrial cancer	MSI status	Internal: TCGA (STAD, CRC; n = 1053)/external: DACHS cohort (n = 378)	ResNet18 (tumor detection + MSI classifier)	AUC: 0.77–0.84 (internal), 0.84 (external), 0.75 (endometrial)
Echle et al. (2020) [12]	Colorectal cancer	MSI status	Internal: TCGA, DACHS, QUASAR, NLCS (N = 7311)/external: YCR-BCIP (N = 2302)	ShuffleNet-based CNN (tile-based DL)	AUC: 0.92 (internal), 0.95–0.96 (external); Biopsy AUC: 0.78 → 0.89 (with biopsy data)
Krause et al. (2021) [13]	Colorectal cancer	MSI status	TCGA (N = 398) + NLCS cohort (n = 1457)	CGAN (image generation) + CNN classifier	AUC: 0.742 (real), 0.743 (synthetic), 0.777 (mixed)
Yamashita et al. (2021) [14]	Colorectal cancer	MSI status	Internal: Stanford (n = 100)/external: TCGA (n = 484)	MobileNetV2-based 2-stage pipeline (MSINet)	AUC: 0.931 (internal), 0.779 (external); NPV 93.7%
Bilal et al. (2021) [15]	Colorectal cancer	Molecular pathways (MSI, CIN, CIMP, hypermutation) + mutations (*BRAF*, *TP53*, *KRAS*)	Internal: TCGA-CRC-DX (n = 502)/external: PAIP cohort (n = 47)	ResNet18 + ResNet34 + HoVer-Net (MIL-like framework)	AUC: MSI 0.86, 0.98 (external); CIN 0.83; CIMP 0.79; hypermutation 0.81; *BRAF* 0.79; *TP53* 0.73; *KRAS* 0.60
Muti et al. (2021) [16]	Gastric cancer	MSI + EBV status (immunotherapy biomarkers)	10 international cohorts (N = 2823 MSI, N = 2685 EBV)	ShuffleNet (tile-based weak supervision, no annotation)	AUC: MSI 0.723–0.863; EBV 0.672–0.859 (external); improved with pooled training
Lee et al. (2021) [17]	Colorectal cancer	MSI status	Internal: TCGA (n = 1336 frozen and n = 584 FFPE)/external: SMH (n = 274 FFPE)	Multi-stage pipeline (tissue filtering + tumor detection + MSI status with Inception-v3)	AUC 0.861–0.942 (TCGA), up to 0.972 (combined dataset), screening potential ~40%
Bustos et al. (2021) [18]	Colorectal cancer	MSI status	Multicenter cohort (EPICOLON + HGUA, N = 1788)	ResNet34-based CNN + adversarial bias-rejecting modules (xDEEP-MSI)	AUC 0.87 (tile-level), 0.90 (patient-level)
Echle et al. (2022) [19]	Colorectal cancer	MSI status	Multicenter cohorts (N = 8343 across 9 cohorts + biopsy cohort N = 1530)	ResNet18-based CNN	AUC: up to 0.96; Rule-out: ~52% at 95% sensitivity
Leiby et al. (2022) [20]	Colorectal cancer	MSI status	Internal: TCGA (N = 360) + external: PAIP (N = 47)	Attention-based MIL + VGG19 + self-supervised contrastive learning	AUC: 0.876 (internal), 0.876 (external)
Jiang et al. (2022) [21]	Colorectal cancer	MMR status (dMMR vs. pMMR)	Internal: TCGA (n = 441)/external: PAIP (n = 78), SYSUCC surgical (n = 355), SYSUCC biopsy (n = 341)	DenseNet-IBN + MIL	AUC: 0.767–0.888
Zhu et al. (2022) [22]	Colorectal + Gastric	MSI status	TCGA-CC-DX (N = 360), TCGA-CC-KR (N = 388), TCGA-STAD (N = 285)	ResNet-18 (DL) + Random Forest (feature-based)	AUC: 0.80–0.84 (DL); 0.70–0.78 (RF)
Schirris et al. (2022) [23]	Colorectal cancer, Breast cancer	MSI, homologous recombination deficiency status	TCGA-CRC (N = 360), TCGA-BC (N = 1041)	Heterogeneity-aware deep MIL (DeepSMILE)	MSI AUC up to 0.87; HRD AUC up to 0.81
Qiu et al. (2022) [24]	Colorectal cancer	MSI status	TCGA (n = 353)	ResNet34 + multi-omics (mRNA, miRNA, lncRNA, methylation, CNV)	AUC: 0.809 (image only), 0.952 (image + DNA methylation)
Su et al. (2022) [25]	Gastric cancer	Differentiation grade + MSI status	Own cohort (n = 467)	ResNet-18 (tile-based CNN + aggregation + Grad-CAM interpretability)	F1 ~0.902 (grading), MSI accuracy ~86.36% (test)
Lou et al. (2022) [26]	Colorectal cancer	MSI status	Multicenter Asian cohort (N = 144, 3 hospitals)	Two-stage DL (tumor detection + pair-wise CNN with partial parameter sharing)	Accuracy 94.29%, AUC 0.942
Ding et al. (2022) [27]	Colorectal cancer	Gene mutations, CNA, MSI, protein expression	Internal: TCGA-COAD (n = 459)/external: TCGA-READ (n = 165), CPTAC-COAD (n = 161)	Spatial GNN (tile graph-based model)	AUC 0.61–0.83 (MSI), 0.70–0.87 (mutations), 0.62–0.90 (CNA), 0.51–0.89 (protein expressions)
Schrammen et al. (2022) [28]	Colorectal cancer	Tumor detection, MSI/dMMR, *BRAF* mutation	Internal: DACHS (N = 2448)/external: YCR-BCIP validation (N = 889)	ShuffleNet-based CNN	Tumor detection AUC 0.980; MSI AUC 0.909 (internal), 0.900 (external); *BRAF* AUC 0.821
Lee et al. (2023) [29]	Gastric cancer	MSI status	Internal: TCGA-STAD (N = 331)/external: SSMH cohort (N = 383)	Inception-v3 (3-stage pipeline: tissue/tumor/MSI)	AUC: 0.893 (frozen), 0.902 (FFPE), 0.874 (external), up to 0.968 (combined datasets)
Rubinstein et al. (2023) [30]	Colon cancer	Tumor heterogeneity + MSI status	TCGA cohort (N = 313)	Inception v3 CNN + clustering + tumor heterogeneity score metric	MSI prediction AUC: ~0.7; THS–MSIsensor correlation r: 0.51
Gerwert et al. (2023) [31]	Colon cancer (early-stage)	MSI status	AIO ColoPredict Plus registry (N = 629 for cancer detection, N = 547 for MSI prediction)	QCL-IR imaging + U-Net (cancer detection) + VGG CNN (MSI prediction)	AUC 0.90 (MSI); sensitivity 0.85; specificity 0.84; cancer detection AUROC 1.0
Niehues et al. (2023) [32]	Colorectal cancer	MSI, mutations (*BRAF*, *KRAS*, *NRAS*, *PIK3CA*)	Internal: QUASAR (N = 2190)/external: DACHS (N = 2448)	self-supervised learning-based encoder + attention-based MIL, optional multi-input	MSI: AUC 0.92–0.94, *BRAF*: 0.82–0.85, others: 0.52–0.67
Guo et al. (2023) [33]	Colorectal cancer	MSI, hypermutation, CIMP, CIN, *BRAF*, *TP53*	Internal: MCO (N = 1065)/external: TCGA (N = 462)	Swin Transformer	AUC MSI 0.904 (external); other biomarkers 0.726–0.847
Yu et al. (2023) [34]	Gastric cancer	MSI status	Internal: Private CGMH dataset (n = 448)/transfer learning: TCGA-STAD	Residual Attention Network + non-local module	AUC up to 0.99, accuracy 96.5% (TCGA)
Wagner et al. (2023) [35]	Colorectal cancer	MSI, *BRAF*/*KRAS* mutation	Multicentric cohort (n > 13,000 patients, 16 cohorts) + external biopsy cohorts	Fully transformer-based model (transformer encoder + transformer aggregation)	MSI: sensitivity 0.99, NPV 0.99; biopsy AUC ~0.92; *BRAF* AUC ~0.88; *KRAS* AUC ~0.80
Zheng et al. (2024) [36]	Gastric cancer	MSI status	Internal: own cohort (n = 1262)/external: multi-center (n = 180), TCGA (n = 284)	ResNet18 + StyleGAN	AUC: 0.930 (internal), 0.895 (external), 0.844 (TCGA)
Gustav et al. (2024) [37]	Colorectal cancer	MSI + POLE mutation (indirect detection)	Internal: DACHS (N = 2039)/external: TCGA (N = 429 + APHP, N = 27 resection, N = 38 biopsy)	Vision Transformer	MSI: AUC 0.94 (internal), 0.87 (external); POLE detection: >75%
Ciardiello et al. (2025) [38]	Gastrointestinal cancers (colorectal, gastric)	MSI status	TCGA-based dataset (192,310 patches, train/val/test split)	CNNs (VGG16, VGG19, Inception, MobileNet) + ViT	Accuracy: ~0.926, AUC: ~0.976
Gustav et al. (2025) [39]	Colorectal cancer	Multi-target biomarker prediction (MSI, *BRAF*, *KRAS*, *RNF43*, *TP53*, *APC*, hypermutation)	Internal: five GECCO cohorts (N = 1376)/external: TCGA (N = 426), CPTAC (N = 110)	Transformer-based (CTransPath features)	MSI AUC 0.93, hypermutation 0.88, *BRAF* 0.78, *RNF43* 0.86
Park et al. (2025) [40]	Colorectal, gastric cancers	MSI status + ICI response prediction	12 datasets (multi-institutional, multi-ethnic): colorectal (n = 2091), gastric (n = 1101)	Deep Gaussian Process + CNN feature extractor	AUC: 0.815–0.953; ICI response accuracy: 94.1%
Aldera et al. (2025) [41]	Colorectal cancer	MSI status	Internal: CPTAC (N = 210) + TCGA (N = 632) + DACHS (N = 2248)/external: South African cohort (n = 197)	Transformer-based + foundation model (Virchow2 + STAMP/COBRA + MIL)	AUC 0.91, Sensitivity 95.6% (calibrated), specificity 69.6% (calibrated)
Zhang et al. (2025) [42]	Colorectal, gastric cancers	MSI, TMB	Internal: TCGA (GC + CRC n = 726/787 for MSI/TMB)/external: CRC cohort (N = 83)	CLAM + Hover-Net + MIL models (feature fusion)	AUC: 0.816–0.834 (MSI internal), 0.81 (MSI external), 0.766–0.800 (TMB internal)
Feng et al. (2025) [43]	Colorectal cancer	MSI status	Internal: 6 cohorts (n = 3736)/external: 1 cohort (n = 1334)	DINOv2 + attention-based MIL	AUC: 0.98, Sensitivity: 95%, specificity: 91%
El Nahhas et al. (2025) [44]	Multi-cancer (including colorectal cancer)	Biomarker prediction (e.g., MSI)	Internal: TCGA/external: CPTAC (MSI prediction use case)	STAMP pipeline (self-supervised feature extractor + transformer-based weakly supervised model)	AUC 0.84 (internal), 0.85 (external), AUPRC 0.58–0.68
Bass et al. (2025) [45]	Colorectal cancer	MSI status	Multi-centre UK cohort (n = 3576)	CNN feature extractor + attention-based aggregation	Definitive results 86.55%, overall agreement 93.83%, positive agreement 92.54%, negative agreement 94.02%
Tang et al. (2025) [46]	Colorectal cancer	MSI status	Internal: Center I and II (n = 254)/external: Center III (n = 51)	EfficientNet-b0 (WSI)/ResNet101 (CT)/adaptive fusion	External AUC Fusion 0.905; pathology-only 0.794; CT-only 0.858
Lee et al. (2025) [47]	Colorectal, gastric, endometrial cancers	MSI status	Internal: TCGA multi-cohort (N = 1244)/external: CPTAC (n = 138)	EfficientNet-b0/ResNet18/VGG19/ConvNeXT	AUC: CRC 0.93/STAD 0.84/UCEC 0.79 (by multi-tissue model)
Yao et al. (2025) [48]	Rectal cancer	MSI status	Internal: one center (n = 347)/external: two centers (n = 120)	ResNet-101 (MRI radiomics + H&E pathomics + clinical integration)	Nomogram (multi-modal) AUC: 0.987 (internal), 0.974 (external)
Salzano et al. (2026) [49]	Colorectal cancer	MMR status (pMMR vs. dMMR)	Retrospective CRC cohort (N = 50)	Vision-language models (ChatGPT-4.0, Gemini 2.5 Pro)	Specificity: 100%, Sensitivity: 65%
Petäinen et al. (2026) [50]	Colorectal cancer	dMMR	Internal: CRC-FFPE-FIN (n = 1228)/external: CRC-OYS-FIN (n = 1010) + TCGA (n = 457)	MobileNetV3/UNI-v2/HOptimus0 for embedding → MIL (ABMIL, CLAM)	External: F1 0.916–0.934; specificity 0.964–0.992; sensitivity 0.500–0.682
Tien et al. (2026) [51]	Colorectal cancer	MSI, *BRAF*/*KRAS* mutation	Internal: TCGA/external: CPTAC	Ensemble MIL (Attention-based + transformer-based + graph-based MIL)	AUC: MSI-H 0.90 (internal)/0.78 (external), *BRAF* 0.87/0.76, *KRAS* 0.64/0.61
Wang et al. (2026) [52]	Gastric cancer	MSI status	TCGA-STAD (N = 282)/TCGA-CRC-DX (N = 357)	ResNet50 + AMIL + MLP + ProMMF_Kron (multimodal fusion)	AUC 0.96 (STAD), 0.99 (CRC)

**Table 3:** Characteristics and performance of studies predicting mutational status from histopathological images.

Reference	Cancer Type	Target	Training/Validation Cohort	Base Model	Performance Measure
Chen et al. (2020) [54]	Hepatocellular carcinoma	Tumor classification, histologic grade, mutation prediction (10 genes)	Internal: TCGA (n = 491)/external: own hospital cohort (n = 101)	Inception-v3 CNN	Classification accuracy: 96.0%/Grading: 89.6%/Mutation AUC: 0.71–0.89
Liao et al. (2020) [55]	Hepatocellular carcinoma	Tumor classification + mutation of 10 genes	Internal: TCGA (n = 491)/external: WCH TMA cohort (n = 455)	ResNet-based CNN	AUC 0.97–1.00 (normal vs. tumor); 0.70–0.90 (mutation)
Jang et al. (2020) [56]	Colorectal cancer	Gene mutations (*APC*, *KRAS*, *PIK3CA*, *SMAD4*, *TP53*)	Internal: TCGA (N = 629)/external: SMH cohort (N = 142)	CNN (Inception-v3, 3-stage pipeline: tissue → tumor → mutation)	AUC 0.645–0.809 (internal), 0.570–0.775 (external), improved with combined training
Bilal et al. (2021) [15]	Colorectal cancer	Molecular pathways (MSI, CIN, CIMP, hypermutation) + mutations (*BRAF*, *TP53*, *KRAS*)	Internal: TCGA-CRC-DX (n = 502)/external: PAIP cohort (n = 47)	ResNet18 + ResNet34 + HoVer-Net (MIL-like framework)	AUC: MSI 0.86, 0.98 (external); CIN 0.83; CIMP 0.79; hypermutation 0.81; *BRAF* 0.79; *TP53* 0.73; *KRAS* 0.60
Bian et al. (2021) [57]	Colorectal cancer	TME cell distribution (CD3/CD20/PanCK/DAPI) + gene mutations (*APC*, *TP53*, *KRAS*)	TCGA-COAD (N = 446 for mutation task)	CBDPN (Inception-v3 + residual + semi-supervised GAN-like) + ShuffleNet V2	Biomarker prediction accuracy 90.4%; mutation AUC: *APC* 0.76, *KRAS* 0.77, *TP53* 0.79
Jang et al. (2021) [58]	Gastric cancer	Gene mutations (*CDH1*, *ERBB2*, *KRAS*, *PIK3CA*, *TP53*)	Internal: TCGA (numbers of patients differ per gene)/external: SSMH cohort	Inception-v3 CNN (patch-based aggregation)	AUC 0.661–0.862 (TCGA), 0.572–0.688 (external), improved with combined datasets
Morel et al. (2022) [59]	Breast, colorectal, lung, and ovarian (pan-cancer)	Mutational status (922 genes, e.g., *TP53*, *KRAS*, *EGFR*)	TCGA (BRCA, LUAD, COAD, OV)	EfficientNetB7-based CNN (patch-level → slide-level aggregation)	AUC: 0.60–0.74 (clinical genes), up to 0.90 (non-clinical genes)
Ghareeb et al. (2022) [60]	Rectal cancer	*KRAS* mutation	Internal: TCGA (49,684 tiles)/external: KUCM cohort (43,032 tiles)	MobileNet, ShuffleNet, Inception (CNNs)	AUC 0.80 (best), sensitivity 91.1%, specificity 65.9%
Jiang et al. (2022) [61]	Colorectal cancer	Tissue type + gene mutations + consensus molecular subtypes (CMS)	Internal: TCGA-COAD (n = 461)/external: PWH cohort (n = 40)	CNN (ResNet-based, multi-task, endoscopy-pretrained)	Tumor vs. normal AUC 0.97; gene mutation AUC 0.7–0.8 (*TP53*, *MYH9*); CMS sensitivity: 0.450–0.727
Ding et al. (2022) [27]	Colorectal cancer	Gene mutations, copy number alterations (CNA), MSI, protein expression	Internal: TCGA-COAD (n = 459)/external: TCGA-READ (n = 165), CPTAC-COAD (n = 161)	Spatial GNN (tile graph-based model)	AUC 0.61–0.83 (MSI), 0.70–0.87 (mutations), 0.62–0.90 (CNA), 0.51–0.89 (protein expressions)
Schrammen et al. (2022) [28]	Colorectal cancer	Tumor detection, MSI/dMMR, *BRAF* mutation	Internal: DACHS (N = 2448)/external: YCR-BCIP validation (N = 889)	ShuffleNet-based CNN (weakly supervised, 3-class: positive/negative/non-informative)	Tumor detection AUC 0.980; MSI AUC 0.909 (internal), 0.900 (external); *BRAF* AUC 0.821
Fu et al. (2023) [62]	Gastrointestinal stromal tumors	Prognosis, gene mutations (*KIT*, *PDGFRA*)	Internal: French cohort 1 (N = 1233)/external: French cohort 2 (n = 286)	CNN (ResNet50, MoCo self-supervised) + MIL-like tile aggregation	Prognosis: C-index 0.72–0.83/mutation: AUC 0.71–0.91
Wei et al. (2023) [63]	Gastric cancer	Immunotherapy biomarkers (EBV, MSI, TMB, PD-L1), gene mutations (*KRAS*, *PIK3CA*, *TP53*, *MUC16*), pathway activity	Internal: TCGA (n = 347) + GasHisSDB (33,284 images)/external: FHCMU cohort (n = 118)	ResNet18 (tumor extraction) + ResNet50 (stratification) + HoverNet (segmentation)	AUC: 0.868–0.946 (immunosensitive subtypes), 0.828–0.922 (mutations), 0.756–0.861 (pathways)
Niehues et al. (2023) [32]	Colorectal cancer	MSI, gene mutations (*BRAF*, *KRAS*, *NRAS*, *PIK3CA*)	Internal: QUASAR (N = 2190)/external: DACHS (N = 2448)	self-supervised learning-based encoder + attention-based MIL, optional multi-input	MSI: AUC 0.92–0.94, *BRAF*: 0.82–0.85, others: 0.52–0.67
Chen et al. (2023) [64]	Colorectal cancer, LUAD, HNSCC	Gene mutations (e.g., *TP53*, *KRAS*, *EGFR*, *PIK3CA*)	TCGA datasets (N ≈ 400 for each cancer)	CNN (Xception feature extraction) + clustering-based MIL	AUC 0.633–0.766 for mutation prediction in CRC
Saldanha et al. (2023) [65]	Pan-cancer (BRCA, CRC, GBM, LUAD, LUSC, PAAD, UCEC)	Gene mutations (OncoKB genes, e.g., *IDH1*, *EGFR*, *TP53*)	Internal: TCGA (7 cancer types)/external: CPTAC (7 cancer types)	ResNet50 (feature extraction) + attention-based MIL	AUC up to 0.84 (*IDH1*), 0.76 (*EGFR*), ~0.7 for multiple genes
Wagner et al. (2023) [35]	Colorectal cancer	MSI, *BRAF*/*KRAS* mutation	Multicentric cohort (n > 13,000 patients, 16 cohorts) + external biopsy cohorts	Fully transformer-based model (transformer encoder + transformer aggregation)	MSI: sensitivity 0.99, NPV 0.99; biopsy AUC ~0.92; *BRAF* AUC ~0.88; *KRAS* AUC ~0.80
Li et al. (2024) [66]	Left-sided colorectal cancer	*KRAS*, *NRAS*, *BRAF* mutation	Chinese cohort (N = 129, 103 train/26 test)	ResNet18/50, Inception v3 + MIL	AUC 0.83, PPV 0.92 in test set
Singh et al. (2024) [67]	Colorectal cancer	*KRAS* mutation	TCGA-CRC-DX (N = 106) + NIB (N = 27) + CRC-5000 (n = 10)	Vision Transformer (XCiT-based, 2-stage pipeline)	AUC 0.691 (TCGA), 0.653 (NIB), accuracy 67–70%
Xiao et al. (2025) [68]	Intrahepatic cholangiocarcinoma	Gene mutations (*FGFR2*, *IDH*, *PBRM1*, *TP53*)	Internal: FAH-SYSU (n = 2069)/external: three other centers (N = 150)	Inception v3 + MIL + self-supervised	AUC: *FGFR2* 0.754 (internal), 0.724 (external); *IDH* 0.713 (internal), 0.656 (external)
Gustav et al. (2025) [39]	Colorectal cancer	Multi-target biomarker prediction (*MSI*, *BRAF*, *KRAS*, *RNF43*, *TP53*, *APC*, hypermutation)	Internal: five GECCO cohorts (N = 1376)/external: TCGA (N = 426), CPTAC (N = 110)	Transformer-based (CTransPath features)	MSI AUC 0.93, hypermutation 0.88, *BRAF* 0.78, *RNF43* 0.86
Zhou et al. (2025) [69]	Colorectal cancer	*BRAF* V600E mutation (IHC-based detection)	Internal: own cohort (250 cases)/external: 31 cases	U-Net (segmentation) + DL-based IHC quantification	AUC 0.938 (cutoff derivation), 0.932 (internal), 0.977 (external)
Tien et al. (2026) [51]	Colorectal cancer	MSI, *BRAF*/*KRAS* mutation	Internal: TCGA/external: CPTAC	Ensemble MIL (Attention-based + transformer-based + graph-based MIL)	AUC: MSI-H 0.90 (internal)/0.78 (external), *BRAF* 0.87/0.76, *KRAS* 0.64/0.61
Bonetti et al. (2026) [70]	Gastrointestinal stromal tumor	*KIT*, *PDGFRA* mutations + treatment sensitivity + recurrence-free survival (RFS)	Multinational cohort (n = 8398, 21 centers, 7 countries)	Foundation model features (CONCH) + Vision Transformer	Mutation AUC 0.87 (*KIT*), 0.96 (*PDGFRA*); treatment AUC 0.81–0.93; RFS HR up to 8.44
Zhang et al. (2026) [71]	Colorectal cancer	*KRAS*, *NRAS*, *BRAF* mutations + HER2 status (IHC)	Single-center cohort (n = 435, internal 10-fold CV)	Vision Transformer (UNI feature extractor) + CLAM	AUC 0.895 (*KRAS*), 0.936 (*NRAS*), 0.987 (*BRAF*), 0.990 (HER2)
Wang et al. (2026) [72]	Hepatocellular carcinoma	Immune score + gene mutations (*CTNNB1*, *TP53*, *CSMD1*)	TCGA-LIHC (N = 370, 5-fold CV + held-out test)	Swin Transformer + Gated Attention MIL	AUC 0.88 (immune score), 0.997 (*CTNNB1*), 0.861 (*TP53*), 0.996 (*CSMD1*)

References [15,27,28,32,35,39,51] are discussed in detail in Section 3.1 (Microsatellite instability).

**Table 4:** Characteristics and performance of studies predicting molecular subtype from histopathological images.

Reference	Cancer Type	Target	Training/Validation Cohort	Base Model	Performance Measure
Liang et al. (2021) [74]	Gastrointestinal stromal tumor	*KIT/PDGFRA* mutation subtype (drug-sensitive vs. others)	365 cases, 5153 images from 3 institutions	AlexNet, ResNet101, DenseNet201, and Inception-v3	Accuracy: up to 87% (DenseNet201)
Hinata et al. (2021) [75]	Gastric adenocarcinoma	Immunotherapy-sensitive subtype (EBV + MSI/dMMR)	Internal: UTokyo cohort (n = 408)/external: TCGA (n = 244)	VGG16-based CNN (fine-tuned)	AUROC: 0.947 (internal), 0.870 (external)
Sirinukunwattana et al. (2021) [76]	Colorectal cancer	Consensus molecular subtypes (CMS)	Internal: FOCUS trial (n = 278)/external: TCGA (n = 430) + GRAMPIAN (n = 144)	Inception-v3 CNN (tile-based, domain-adversarial training)	AUC 0.80–0.85 across cohorts
Chen et al. (2021) [77]	Gastric cancer	Immune subtypes (based on tumor-infiltrating immune cells)	TCGA (n = 194) + GEO (n = 299)	ResNet-18	slide-level accuracy (test set): ~76%
Zheng et al. (2022) [78]	Gastric cancer	EBV status	Internal: Internal-STAD (n = 1006)/external: MultiCenter-STAD (n = 417) + TCGA-STAD (n = 234)	ResNet50-based CNN	AUROC: 0.969 (internal), 0.941 (multi-center), 0.895 (TCGA)
Jeong et al. (2022) [79]	Gastric cancer	EBV status	Internal: TCGA (n = 319) + ISH (n = 108)/external: HGH (n = 60)	ResNet50 (tumor) + Inception-v3 (EBV)	AUC: 0.88; AUPRC: 0.65; NPV 0.98; sensitivity 0.86
Vuong et al. (2022) [80]	Gastric cancer	EBV status	TMAs (n = 16) + resection (n = 24) + biopsy (n = 286)	EfficientNet (patch-based classification)	Patch-level accuracy 94.7%, biopsy-level AUC 0.872
Flinner et al. (2022) [81]	Gastric adenocarcinoma	TCGA molecular subtypes (EBV, MSI, GS, CIN)	Internal: TCGA (n = 133)/external: UKC cohort (n = 65)	Ensemble CNN (DenseNet161, bagging)	AUC ~0.76 (4-class, patient-level)
Wang et al. (2022) [82]	Gastric adenocarcinoma	TCGA molecular subtypes (EBV, MSI, GS, CIN)	TCGA (n = 332)	EfficientNet-b1 ensemble	Patient-level AUC 0.764–0.898
Jiang et al. (2022) [61]	Colorectal cancer	Tissue type + gene mutations + CMS	Internal: TCGA-COAD (n = 461)/external: PWH cohort (n = 40)	CNN (ResNet-based, multi-task, endoscopy-pretrained)	Tumor vs. normal AUC 0.97; gene mutation AUC 0.7–0.8 (TP53, MYH9); CMS sensitivity: 0.450–0.727
Saillard et al. (2023) [83]	Pancreatic ductal adenocarcinoma	Molecular subtype (basal-like vs. classical) + stromal subtype (active vs. inactive)	Internal: DISC (n = 424)/external: multicenter cohorts including TCGA (n = 1796)	Multi-step DL pipeline (tile-based CNN features → sequential models: tumor detection + cell-type + subtype prediction)	AUC 0.81–0.86 (external), improved up to 0.91–0.95 in homogeneous cases
Ahmadvand et al. (2024) [84]	Pancreatic ductal adenocarcinoma	Molecular subtypes (classical vs. basal-like)	Internal: TCGA-PAAD (n = 97)/external: POG-PANGEN biopsy samples (n = 110)	ResNet-based CNN + MIL variants	Accuracy: 96.19% (internal), 83.03% (external); AUC: ~0.867
Hyeon et al. (2024) [85]	Colorectal cancer	CIN (Aneuploidy score)	TCGA (n = 315)	Tile-based CNN feature + nucleus-based morphologic feature	AUC 0.75
Lafarge et al. (2024) [86]	Rectal cancer	CMSs (CMS1–4) + treatment response	Internal: Multicentric cohort (n = 1057)/external: independent cohorts (n = 169)	ResNet-based CNN (tile-level → slide aggregation, imCMS)	macro-average AUC 0.813; treatment response: OR 2.69 (imCMS1), OR 0.25 (imCMS4)
Byeon et al. (2025) [87]	Gastric cancer	EBV status	Internal: KSH (n = 2684), external: TCGA (n = 347)	EfficientNet + stain normalization	AUC: 0.66–0.84 (internal), 0.72–0.77 (external)
Kaprio et al. (2025) [88]	Colorectal cancer	CMS-like molecular subtype based on IHC (CMS1–4)	IHC-based cohort (n = 538)	CNN-assisted IHC classifier	Classification success rate: 89.4%
Seraphin et al. (2025) [89]	Hepatocellular carcinoma	Molecular subclass (S1/S2 vs. S3), microvascular invasion (mVI)	Internal: own cohort (n = 431)/external: TCGA (n = 363) + advanced HCC (n = 64)	Transformer-based MIL (UNI features)	Molecular subclass AUC: 0.72–0.81; mVI AUC: 0.62
Le Douget et al. (2025) [90]	Colon cancer	CMSs (CMS1–4)	Internal: PETACC-8 (n = 1341) + TCGA (n = 384)/external: PRODIGE-13 (n = 407)	iBOT ViT (feature) + AB-MIL	AUC: 0.92–0.94 (external)
Akbar et al. (2026) [91]	Pancreatic ductal adenocarcinoma	Molecular subtype (basal-like vs. classical)	Internal: PANCAN (n = 846)/external: TCGA (n = 209)	CellViT++ + UNI2-h + multi-scale fusion + attention MIL	AUC 0.88 (internal), 0.84 (external)

Reference [61] is discussed in detail in Section 3.2 (Mutational status).

**Table 5:** Characteristics and performance of studies predicting treatment response or prognostic biomarker from histopathological images.

Reference	Cancer Type	Target	Training/Validation Cohort	Base Model	Performance Measure
Zhao et al. (2020) [92]	Colorectal cancer	Tumour/stroma ratio → overall survival (OS) prediction	Internal: (N = 499)/external: (N = 315)	CNN (VGG-19, tissue segmentation)	C-index: 0.721; hazard ratio (HR): 1.72–2.08
Patil et al. (2023) [93]	Hepatocellular carcinoma	Reticulin proportionate area → metastasis, disease free survival (DFS), OS	Single center resection cohort (101 cases)	Supervised DL (Aiforia platform)	HR: 3.8 (metastasis), 2.5 (DFS), 2.8 (OS)
Makhlouf et al. (2023) [94]	Colorectal cancer + pan-cancer validation	CD3, CD4, CD8 (T-cell density)	Internal: multicentric dataset (N = 1041)/external: pan-cancer (13 tumor types) for CD3 density	U-Net (T-cell segmentation) + ResNet-34 (feature encoder) + survival model (Cox/KM)	Segmentation Dice up to 70.31%; survival stratification *p* = 0.002; CD3 cross-tumor correlation ~0.90
Zeng et al. (2023) [95]	Hepatocellular carcinoma	atezolizumab–bevacizumab response gene signature → PFS	Internal: TCGA (N = 336)/external: cohort1 (n = 225), cohort2 (n = 157), treatment cohort: (N = 122)	CLAM-based MIL + CTransPath	Pearson’s coefficient r: 0.53–0.86/PFS: 12 vs. 7 months
Wei et al. (2023) [63]	Gastric cancer	Immunotherapy biomarkers (EBV, MSI, TMB, PD-L1), gene mutations (*KRAS*, *PIK3CA*, *TP53*, *MUC16*), pathway activity	Internal: TCGA (n = 347) + GasHisSDB (33,284 images)/external: FHCMU cohort (n = 118)	ResNet18 (tumor extraction) + ResNet50 (stratification) + HoverNet (segmentation)	AUC: 0.868–0.946 (immunosensitive subtypes), 0.828–0.922 (mutations), 0.756–0.861 (pathways)
Nimgaonkar et al. (2023) [96]	Pancreatic ductal adenocarcinoma	Gemcitabine treatment response	Internal: TCGA (N = 93)/external: UPMC (n = 46), untreated Copenhagen (n = 161)	HoVer-Net (nuclei segmentation) + handcrafted morphologic features + LASSO Cox model	Significant DSS stratification (HR 2.94)
Horst et al. (2023) [97]	Esophageal/gastroesophageal junction adenocarcinoma	Response to neoadjuvant chemotherapy (PET/CT-defined)	MEMORI multicenter cohort (N = 67)	CNN (ResNet50), ViT + CLAM	Pretherapy AUC 0.81, On-therapy AUC 0.84, balanced accuracy 0.78–0.80
Tian et al. (2024) [98]	Hepatocellular carcinoma	M2 macrophage abundance → prognosis	TCGA (353 patients) + own hospital (132 patients)	Masked autoencoders (self-supervised) + ResNet-32t	AUC: 0.73 for M2 abundance; prognostic HR: up to 2.36
Liu et al. (2024) [99]	Gastric cancer (advanced, unresectable)	PD-1 inhibitor + chemotherapy response	Internal: FAH-SYSU (N = 139)/external: 3 other centers (N = 125)	Ensemble (EfficientNet-B4 + DenseNet121 + Swin Transformer V2)	AUC: 0.92–1.00 (external), 0.952 (internal)
Lafarge et al. (2024) [86]	Rectal cancer	Consensus molecular subtype (CMS1–4) + treatment response	Multicentric cohort (n = 1057) + independent test cohorts (n = 169)	ResNet-based CNN (tile-level → slide aggregation, imCMS)	macro-average AUC 0.813; treatment response: OR 2.69 (imCMS1), OR 0.25 (imCMS4)
Kim et al. (2025) [100]	Biliary tract cancer (HER2+)	HER2 status (IHC), immune phenotype (TIL spatial analysis) → PFS, OS, treatment response	Phase II trial cohort (n = 29)	Lunit SCOPE HER2 (DeepLab v3+ and ResNet-34)	Concordance with pathologists: 79.1%; PFS HR ~0.40; OS HR ~0.19
Kong et al. (2025) [101]	Gastrointestinal stromal tumors	TKI response (imatinib, avapritinib)	Internal: training cohort (n = 747), external: test cohort (61 cases)	CNN (ResNet50) + Transformer-based MIL	AUC: ~0.958, accuracy: ~0.9286
Park et al. (2025) [40]	Colorectal + gastric cancers	MSI status + immune checkpoint inhibitor response prediction	12 datasets (multi-institutional, multi-ethnic): colorectal (n = 2091), gastric (n = 1101)	Deep Gaussian Process + CNN feature extractor	AUC: 0.815–0.953; ICI response accuracy: 94.1%
Wu et al. (2025) [102]	Gastric adenocarcinoma	HER2 status (H&E) + trastuzumab response	Surgical (n = 300) + biopsy (n = 101) + treatment cohort (n = 41)	ResNet50 + CLAM	HER2: AUC 0.847 (surgical), 0.903 (2+), 0.723 (biopsy); trastuzumab response: AUC ~0.833
Zhu et al. (2026) [103]	Esophageal squamous cell carcinoma	Neoadjuvant chemoimmunotherapy response	Tongji cohort (N = 104 train, N = 74 validation)) + TCGA (94 cases) + GSE160269 (64 cases)	ResNet152 (segmentation) + clinical integration → pathomic signature score	AUC for major pathologic response 0.897 (train), 0.809 (validation)
Hendifar et al. (2026) [104]	Pancreatic ductal adenocarcinoma (advanced)	Chemotherapy selection biomarker	Internal: Cedars and UPMC cohorts (N = 178)/external: COMPASS and KYT cohorts (N = 299)	CHAI platform (histomorphologic feature extraction + Cox model)	time to next treatment or death *p* < 0.05 between groups
Bonetti et al. (2026) [70]	Gastrointestinal stromal tumor	*KIT*, *PDGFRA* mutations + treatment sensitivity + recurrence-free survival (RFS)	Multinational cohort (n = 8398, 21 centers, 7 countries)	Foundation model features (CONCH) + Vision Transformer	Mutation AUC 0.87 (*KIT*), 0.96 (*PDGFRA*); treatment AUC 0.81–0.93; RFS HR up to 8.44

Reference [40] is discussed in detail in Section 3.1 (Microsatellite instability); References [63,70] are discussed in detail in Section 3.2 (Mutational status); and Reference [86] is discussed in detail in Section 3.3 (Molecular subtype).

**Table 6:** Characteristics and performance of studies predicting tumor mutational burden from histopathological images.

Reference	Cancer Type	Target	Training/Validation Cohort	Base Model	Performance Measure
Zhang et al. (2019) [106]	Hepatocellular carcinoma	Tumor mutational burden (TMB-H vs. TMB-L)	TCGA (n = 380)	CNN with modified receptive field	Patch-level accuracy: 0.948, AUC: 0.948; patient-level accuracy: 0.997
Wang et al. (2020) [107]	Colorectal + gastric cancers	Tumor mutational burden (TMB-H vs. TMB-L)	TCGA-STAD (280 patients), TCGA-COAD (265 patients)	CNN transfer learning + MIL	AUC: 0.75 (STAD), 0.82 (COAD); Accuracy: up to 0.77
Shimada et al. (2021) [108]	Colorectal cancer	TMB-high (immunotherapy biomarker)	Japanese cohort (N = 201) + TCGA (N = 77)	Inception-v3 (tile-based pipeline)	AUC ~0.943 (average), up to 0.981
Huang et al. (2022) [109]	Colorectal cancer	Tumor mutational burden (TMB-H vs. TMB-L)	TCGA cohort (N = 509)	ResNet18 + multimodal fusion (image + clinical features)	AUC 0.817, accuracy 0.872 (multimodal)
Liu et al. (2022) [110]	Colorectal cancer	Tumor mutational burden (immunotherapy biomarker)	TCGA cohort (N = 509)	ResNet50 (DeepHE framework)	AUC ~0.73 (cutoff = 10), ~0.77 (cutoff = 20)
Li et al. (2024) [111]	Gastric cancer	Tumor mutational burden (TMB-H vs. TMB-L)	TCGA cohort (N = 326)	ResNet18 + multimodal fusion (image + clinical + multi-omics)	AUC 0.749 (image only), AUC 0.971 (image + mRNA)
Zhang et al. (2025) [42]	Colorectal + gastric cancers	MSI, tumor mutational burden	Internal: TCGA (GC + CRC n = 726/787 for MSI/TMB)/external: CRC cohort (N = 83)	CLAM + Hover-Net + MIL models (feature fusion)	AUC: 0.816–0.834 (MSI internal), 0.81 (MSI external), 0.766–0.800 (TMB internal)
Wang et al. (2025) [112]	Colorectal and endometrial cancer	Tumor mutational burden + subtypes	TCGA CRC (n = 1495)/TCGA EC (n = 918)	DINO-ViT + Transformer MIL + Ensemble	AUROC: 0.90 (CRC TMB), 0.87 (CRC subtype), 0.83 (EC TMB), 0.92 (EC subtype)

Reference [42] is discussed in detail in Section 3.1 (Microsatellite instability).

**Table 7:** Characteristics and performance of studies predicting immunohistochemistry-based biomarker from histopathological images.

Reference	Cancer Type	Target	Training/Validation Cohort	Base Model	Performance Measure
Sarker et al. (2021) [113]	Colorectal cancer	ICOS protein (IHC biomarker)	Epi700 cohort (TMA)	U-Net (EfficientNetB7)	Dice coefficient: ~0.73, Jaccard Index: ~0.59
Han et al. (2022) [114]	Gastric cancer	HER2 status (IHC)	Single-center (Fujian Cancer Hospital, n = 183)	RepVGG-based CNN + MLP/SVM (WSI-level aggregation)	WSI-level accuracy up to 94%
Wang et al. (2025) [115]	Gastrointestinal cancers (esophageal, gastric, colorectal)	Multi-IHC biomarker prediction (P40, Pan-CK, Desmin, P53, and Ki-67)	Internal: WURH cohort (n = 134)/external: (n = 150)	ResNet-50 (mean teacher semi-supervised framework)	AUROC 0.90–0.96, accuracy 83–91%
Liao et al. (2025) [116]	Gastric cancer	HER2 status (H&E)	Internal: own cohort (n = 646)/external: multicenter cohort (n = 102)	SegNet (tumor detector) + ResNet50 + RF	Accuracy: 0.904 (internal), 0.892 (external); AUROC: up to 0.9898
Kim et al. (2025) [100]	Biliary tract cancer (HER2+)	HER2 status (IHC), immune phenotype (TIL spatial analysis) → PFS, OS, treatment response	Phase II trial cohort (n = 29)	Lunit SCOPE HER2 (DeepLab v3+ and ResNet-34)	Concordance with pathologists: 79.1%; PFS HR ~0.40; OS HR ~0.19
Wu et al. (2025) [102]	Gastric adenocarcinoma	HER2 status (H&E) + trastuzumab response	Surgical (n = 300) + biopsy (n = 101) + treatment cohort (n = 41)	ResNet50 + CLAM	HER2: AUC 0.847 (surgical), 0.903 (2+), 0.723 (biopsy); Trastuzumab response: AUC ~0.833
Fischer et al. (2026) [117]	Pancreatic ductal adenocarcinoma	*KRT81*, *HNF1A* subtype	140 patients for training, 20 patients for validation	Contrastive CycleGAN + MIL, ResNet/UNI2 for feature extraction	F1: 0.73–0.77 (virtual IHC) vs. 0.84–0.87 (real IHC)
Zhang et al. (2026) [71]	Colorectal cancer	*KRAS*, *NRAS*, *BRAF* mutations + HER2 status (IHC)	Single-center cohort (n = 435, internal 10-fold CV)	ViT (UNI feature extractor) + CLAM	AUC 0.895 (*KRAS*), 0.936 (*NRAS*), 0.987 (*BRAF*), 0.990 (*HER2*)

Reference [71] is discussed in detail in Section 3.2 (Mutational status), and References [100,102] are discussed in detail in Section 3.4 (Treatment response or prognostic biomarker).

**Table 8:** Characteristics and performance of studies predicting gene expression or transcriptomic signature/other biomarkers from histopathological images.

Reference	Cancer Type	Target	Training/Validation Cohort	Base Model	Performance Measure
Schmauch et al. (2020) [119]	Pan-cancer (28 types)	RNA-Seq gene expression (30,000+ genes), MSI status (downstream)	TCGA (n = 10,514)	HE2RNA (ResNet50-based CNN + MLP)	Gene-level correlation: 0.11–0.47; MSI prediction AUC: up to 0.81
Zeng et al. (2022) [120]	Hepatocellular carcinoma	Immune/inflammatory gene signatures (6 signatures)	Internal: TCGA (N = 336)/external own cohort (N = 139)	Patch-based, MIL, CLAM	AUC: 0.78–0.91 (internal), 0.81–0.92 (external)
El Nahhas et al. (2024) [121]	Pan-cancer (9 types: BRCA, CRC, LUAD, etc.)	Homologous recombination deficiency, immune/stromal signatures	Internal: TCGA/external: CPTAC & DACHS/total (n = 11,671)	ResNet50 + attention MIL (CAMIL regression)	AUC 0.62–0.96 (external), improved vs. classification; better Pearson r
Nonchev et al. (2025) [122]	Colon, Kidney, Lung cancers, Melanoma	Spatial transcriptomics (gene expression prediction)	Visium datasets (56 samples)/TCGA (1792 samples)	Deep-set neural network	Pearson correlation up to 0.70
Nonchev et al. (2025) [123]	Lung, Breast, Pancreatic cancers	Single-cell spatial transcriptomics (gene expression prediction)	Xenium dataset (20 lung, 7 breast, 2 pancreas samples)	Deep-set neural network	Pearson correlation 0.39–0.43
Aalam et al. (2025) [124]	Esophageal cancer	Metastasis + oncogenic pathway prediction (mTOR, p53, PI3K/AKT, PTEN)	Internal: TCGA-ESCA (N = 124)/external: TCGA-STAD (N = 20)	CNN (ResNet101 feature extraction) + ML classifiers (KNN, AdaBoost, DT)	AUC 0.92 (metastasis), 0.64–0.92 (oncogenic pathway)

## Data Availability

No new data were generated or analyzed in this study. This article is based on the previously published literature, and therefore data sharing is not applicable.

## Supplementary Materials

The following supporting information can be downloaded at: https://www.mdpi.com/article/10.3390/ijms27156969/s1.

## Abbreviations

The following abbreviations are used in this manuscript:

AI	Artificial intelligence
AUC	Area under the receiver operating characteristic curve
CNA	Copy number alterations
CNN	Convolutional neural network
CRC	Colorectal cancer
CV	Cross-validation
DFS	Disease-free survival
DL	Deep learning
DSS	Disease-specific survival
EBV	Epstein–Barr virus
EC	Endometrial cancer
GC	Gastric cancer
H&E	Hematoxylin and eosin
HCC	Hepatocellular carcinoma
HR	Hazard ratio
HRD	Homologous recombination deficiency
ICI	Immune checkpoint inhibitor
IHC	Immunohistochemistry
MIL	Multiple-instance learning
MMR	Mismatch repair
MSI	Microsatellite instability
MSI-H	Microsatellite instability high
MSS	Microsatellite stable
mVI	Microvascular invasion
NGS	Next-generation sequencing
OS	Overall survival
PCR	Polymerase chain reaction
PFS	Progression-free survival
RFS	Recurrence-free survival
RPA	Reticulin proportionate area
THS	Tumor heterogeneity score
TKI	Tyrosine kinase inhibitor
TMB	Tumor mutational burden
TME	Tumor microenvironment
TSR	Tumor/stroma ratio
ViT	Vision transformer
WSI	Whole-slide image
