# Supplementary material for: Artificial Intelligence for Molecular Biomarker Identification in Gastrointestinal and Hepatobiliary Cancers
Source: Int J Mol Sci. 2026 Aug 3;27(15):6969. doi: 10.3390/ijms27156969 (PMC13467377; doi:10.3390/ijms27156969)
Supplement: Supplementary file 1 [file ijms-27-06969-s001.zip › Supplementary Figure S1.pdf]

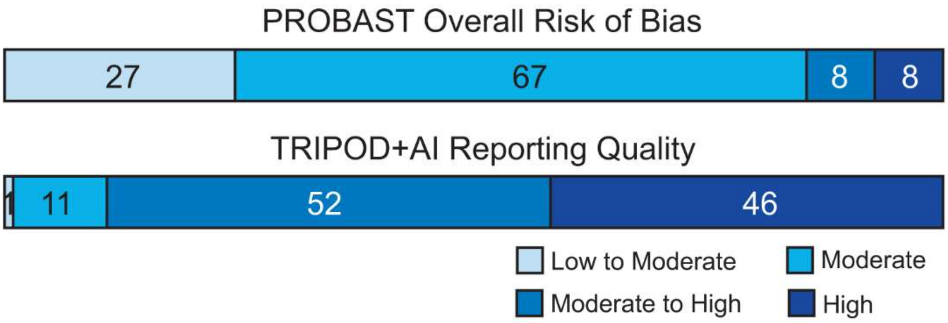

Supplementary Figure S1. Distribution of included studies according to overall risk-of-bias and reporting-quality categories assessed using PROBAST and TRIPOD+AI, respectively.
