# Supplementary material for: Artificial Intelligence for Molecular Biomarker Identification in Gastrointestinal and Hepatobiliary Cancers
Source: Int J Mol Sci. 2026 Aug 3;27(15):6969. doi: 10.3390/ijms27156969 (PMC13467377; doi:10.3390/ijms27156969)
Supplement: Supplementary file 1 [file ijms-27-06969-s001.zip › Supplementary Methods-File S1.pdf]

The search strategies used for each database are provided below.

<PubMed Search Strategy>

(

"Digestive System Neoplasms"[Mesh]

OR "Gastrointestinal Neoplasms"[Mesh]

OR "Colorectal Neoplasms"[Mesh]

OR "Stomach Neoplasms"[Mesh]

OR "Liver Neoplasms"[Mesh]

OR "Carcinoma, Hepatocellular"[Mesh]

OR "Biliary Tract Neoplasms"[Mesh]

OR "Cholangiocarcinoma"[Mesh]

OR "Pancreatic Neoplasms"[Mesh]

OR gastrointestinal cancer*[tiab]

OR gastrointestinal neoplasm*[tiab]

OR digestive cancer*[tiab]

OR digestive neoplasm*[tiab]

OR colorectal cancer*[tiab]

OR colon cancer*[tiab]

OR rectal cancer*[tiab]

OR gastric cancer*[tiab]

OR stomach cancer*[tiab]

OR liver cancer*[tiab]

OR hepatic cancer*[tiab]

OR hepatocellular carcinoma[tiab]

OR HCC[tiab]

OR biliary tract cancer*[tiab]

OR cholangiocarcinoma[tiab]

OR gallbladder cancer*[tiab]

OR pancreatic cancer*[tiab]

OR pancreas cancer*[tiab]

)

AND

(

"Pathology"[Mesh]

OR "Histology"[Mesh]

OR "Whole Slide Imaging"[Supplementary Concept]

OR pathology[tiab]

OR pathologic[tiab]

OR pathological[tiab]

OR histopatholog*[tiab]

OR histolog*[tiab]

OR "whole slide imag*"[tiab]

OR WSI[tiab]

OR "digital pathology"[tiab]

OR "pathology slide*"[tiab]

OR "pathologic slide*"[tiab]

OR "histology slide*"[tiab]

)

AND

(

"Artificial Intelligence"[Mesh]

OR "Machine Learning"[Mesh]

OR "Neural Networks, Computer"[Mesh]

OR "Image Interpretation, Computer-Assisted"[Mesh]

OR "deep learning"[tiab]

OR "artificial intelligence"[tiab]

OR AI[tiab]

OR "machine learning"[tiab]

OR "neural network*"[tiab]

OR CNN[tiab]

OR "convolutional neural network*"[tiab]

OR transformer*[tiab]

OR "foundation model*"[tiab]

)

AND

(

"Biomarkers, Tumor"[Mesh]

OR "Biomarkers"[Mesh]

OR "Mutation"[Mesh]

OR "Gene Expression Profiling"[Mesh]

OR "Gene Expression"[Mesh]

OR "Transcriptome"[Mesh]
OR "Genomics"[Mesh]
OR biomarker*[tiab]
OR molecular[tiab]
OR mutation*[tiab]
OR genomic*[tiab]
OR transcriptomic*[tiab]
OR "gene expression"[tiab]
OR "molecular subtype*"[tiab]
OR MSI[tiab]
OR "microsatellite instability"[tiab]
OR KRAS[tiab]
OR TP53[tiab]
OR BRAF[tiab]
OR HER2[tiab]
)
NOT
(
review[pt]
OR editorial[pt]
OR comment[pt]
OR letter[pt]
)
AND english[lang]
AND ("2015/01/01"[Date - Publication] : "3000"[Date - Publication])

<Embase Search Strategy>

(
'digestive system tumor'/exp
OR 'gastrointestinal tumor'/exp
OR 'colorectal tumor'/exp
OR 'stomach tumor'/exp
OR 'liver tumor'/exp
OR 'hepatocellular carcinoma'/exp
OR 'biliary tract tumor'/exp
OR 'cholangiocarcinoma'/exp

```
    OR 'pancreas tumor'/exp
    OR 'gastrointestinal cancer*':ti,ab,kw
    OR 'gastrointestinal neoplasm*':ti,ab,kw
    OR 'digestive cancer*':ti,ab,kw
    OR 'colorectal cancer*':ti,ab,kw
    OR 'colon cancer*':ti,ab,kw
    OR 'rectal cancer*':ti,ab,kw
    OR 'gastric cancer*':ti,ab,kw
    OR 'stomach cancer*':ti,ab,kw
    OR 'liver cancer*':ti,ab,kw
    OR 'hepatocellular carcinoma':ti,ab,kw
    OR hcc:ti,ab,kw
    OR 'biliary tract cancer*':ti,ab,kw
    OR cholangiocarcinoma:ti,ab,kw
    OR 'gallbladder cancer*':ti,ab,kw
    OR 'pancreatic cancer*':ti,ab,kw
)
AND
(
    'pathology'/exp
    OR 'histopathology'/exp
    OR 'digital pathology':ti,ab,kw
    OR pathology:ti,ab,kw
    OR pathologic*:ti,ab,kw
    OR histopatholog*:ti,ab,kw
    OR histolog*:ti,ab,kw
    OR 'whole slide imag*':ti,ab,kw
    OR wsi:ti,ab,kw
    OR 'pathology slide*':ti,ab,kw
    OR 'pathologic slide*':ti,ab,kw
)
AND
(
    'artificial intelligence'/exp
    OR 'machine learning'/exp
    OR 'deep learning'/exp
```

OR 'neural network'/exp

OR 'image analysis'/exp

OR 'artificial intelligence':ti,ab,kw

OR ai:ti,ab,kw

OR 'machine learning':ti,ab,kw

OR 'deep learning':ti,ab,kw

OR 'neural network*':ti,ab,kw

OR cnn:ti,ab,kw

OR 'convolutional neural network*':ti,ab,kw

OR transformer*:ti,ab,kw

OR 'foundation model*':ti,ab,kw

)

AND

(

'biological marker'/exp

OR 'tumor marker'/exp

OR 'gene expression'/exp

OR 'mutation'/exp

OR 'genomics'/exp

OR 'transcriptomics'/exp

OR biomarker*:ti,ab,kw

OR molecular:ti,ab,kw

OR mutation*:ti,ab,kw

OR genomic*:ti,ab,kw

OR transcriptomic*:ti,ab,kw

OR 'gene expression':ti,ab,kw

OR 'molecular subtype*':ti,ab,kw

OR msi:ti,ab,kw

OR 'microsatellite instability':ti,ab,kw

OR kras:ti,ab,kw

OR tp53:ti,ab,kw

OR braf:ti,ab,kw

OR her2:ti,ab,kw

)

NOT

(

```
  'review'/it
  OR 'editorial'/it
  OR 'letter'/it
  OR 'conference abstract'/it
)
AND [english]/lim
AND [2015-2026]/py
```

<Web of Science Search Strategy>

```
TS=(
  (
    "gastrointestinal cancer*" OR "gastrointestinal neoplasm*" OR
    "digestive cancer*" OR "digestive neoplasm*" OR
    "colorectal cancer*" OR "colon cancer*" OR "rectal cancer*" OR
    "gastric cancer*" OR "stomach cancer*" OR
    "liver cancer*" OR "hepatic cancer*" OR "hepatocellular carcinoma" OR HCC OR
    "biliary tract cancer*" OR cholangiocarcinoma OR "gallbladder cancer*" OR
    "pancreatic cancer*" OR "pancreas cancer*"
  )
  AND
  (
    pathology OR pathologic* OR pathological OR histopatholog* OR histolog* OR
    "whole slide imag*" OR WSI OR "digital pathology" OR
    "pathology slide*" OR "pathologic slide*"
  )
  AND
  (
    "artificial intelligence" OR AI OR
    "machine learning" OR "deep learning" OR
    "neural network*" OR CNN OR "convolutional neural network*" OR
    transformer* OR "foundation model*"
  )
  AND
  (
    biomarker* OR molecular OR mutation* OR genomic* OR transcriptomic* OR
    "gene expression" OR "molecular subtype*" OR
```

```
        MSI OR "microsatellite instability" OR KRAS OR TP53 OR BRAF OR HER2
    )
)
NOT TS=(radiology OR CT OR MRI OR ultrasound)
AND LANGUAGE: (English)
AND PY=(2015-2026)
NOT DT=(Review OR Editorial Material OR Letter)
```

<Scopus Search Strategy>

```
TITLE-ABS-KEY(
    (
        "gastrointestinal cancer*" OR "gastrointestinal neoplasm*" OR
        "digestive cancer*" OR "digestive neoplasm*" OR
        "colorectal cancer*" OR "colon cancer*" OR "rectal cancer*" OR
        "gastric cancer*" OR "stomach cancer*" OR
        "liver cancer*" OR "hepatic cancer*" OR "hepatocellular carcinoma" OR HCC OR
        "biliary tract cancer*" OR cholangiocarcinoma OR "gallbladder cancer*" OR
        "pancreatic cancer*" OR "pancreas cancer*"
    )
    AND
    (
        pathology OR pathologic* OR pathological OR histopatholog* OR histolog* OR
        "whole slide imag*" OR WSI OR "digital pathology" OR
        "pathology slide*" OR "pathologic slide*"
    )
    AND
    (
        "artificial intelligence" OR AI OR
        "machine learning" OR "deep learning" OR
        "neural network*" OR CNN OR "convolutional neural network*" OR
        transformer* OR "foundation model*"
    )
    AND
    (
        biomarker* OR molecular OR mutation* OR genomic* OR transcriptomic* OR
        "gene expression" OR "molecular subtype*" OR
```

MSI OR "microsatellite instability" OR KRAS OR TP53 OR BRAF OR HER2

)

)

AND PUBYEAR > 2014

AND (LIMIT-TO(LANGUAGE, "English"))

AND NOT (DOCTYPE, "re")

AND NOT (DOCTYPE, "ed")

AND NOT (DOCTYPE, "le")

<IEEE Xplore Search Strategy>

(

(

"gastrointestinal cancer" OR "gastrointestinal cancers" OR

"gastrointestinal neoplasm" OR "gastrointestinal neoplasms" OR

"digestive cancer" OR "digestive cancers" OR

"digestive neoplasm" OR "digestive neoplasms" OR

"colorectal cancer" OR "colorectal cancers" OR

"colon cancer" OR "colon cancers" OR

"rectal cancer" OR "rectal cancers" OR

"gastric cancer" OR "gastric cancers" OR

"stomach cancer" OR "stomach cancers" OR

"liver cancer" OR "liver cancers" OR

"hepatic cancer" OR "hepatic cancers" OR

"hepatocellular carcinoma" OR HCC OR

"biliary tract cancer" OR "biliary tract cancers" OR

cholangiocarcinoma OR cholangiocarcinomas OR

"gallbladder cancer" OR "gallbladder cancers" OR

"pancreatic cancer" OR "pancreatic cancers" OR

"pancreas cancer" OR "pancreas cancers"

)

AND

(

pathology OR pathologic OR pathological OR

histopathology OR histopathologies OR

histology OR histologies OR

"whole slide image" OR "whole slide images" OR

```
    WSI OR
    "digital pathology" OR
    "pathology slide" OR "pathology slides" OR
    "pathologic slide" OR "pathologic slides"
  )
  AND
  (
    "artificial intelligence" OR AI OR
    "machine learning" OR
    "deep learning" OR
    "neural network" OR "neural networks" OR
    CNN OR
    "convolutional neural network" OR "convolutional neural networks" OR
    transformer OR transformers OR
    "foundation model" OR "foundation models"
  )
  AND
  (
    biomarker OR biomarkers OR
    molecular OR
    mutation OR mutations OR
    genomic OR genomics OR
    transcriptomic OR transcriptomics OR
    "gene expression" OR
    "molecular subtype" OR "molecular subtypes" OR
    MSI OR
    "microsatellite instability" OR
    KRAS OR TP53 OR BRAF OR HER2
  )
)
NOT
(
  radiology OR CT OR MRI OR ultrasound
)
```
